# Information Inequalities via Submodularity and a Problem in Extremal Graph Theory

**DOI:** 10.3390/e24050597

**Published:** 2022-04-25

**Authors:** Igal Sason

**Affiliations:** 1 Andrew & Erna Viterbi Faculty of Electrical and Computer Engineering, Technion-Israel Institute of Technology, Haifa 3200003, Israel; eeigal@technion.ac.il; Tel.: +972-4-8294699; 2 Faculty of Mathematics, Technion-Israel Institute of Technology, Haifa 3200003, Israel

**Keywords:** extremal combinatorics, graphs, Han’s inequality, information inequalities, polymatroid, rank function, set function, Shearer’s lemma, submodularity

## Abstract

The present paper offers, in its first part, a unified approach for the derivation of families of inequalities for set functions which satisfy sub/supermodularity properties. It applies this approach for the derivation of information inequalities with Shannon information measures. Connections of the considered approach to a generalized version of Shearer’s lemma, and other related results in the literature are considered. Some of the derived information inequalities are new, and also known results (such as a generalized version of Han’s inequality) are reproduced in a simple and unified way. In its second part, this paper applies the generalized Han’s inequality to analyze a problem in extremal graph theory. This problem is motivated and analyzed from the perspective of information theory, and the analysis leads to generalized and refined bounds. The two parts of this paper are meant to be independently accessible to the reader.

## 1. Introduction

Information measures and information inequalities are of fundamental importance and wide applicability in the study of feasibility and infeasibility results in information theory, while also offering very useful tools which serve to deal with interesting problems in various fields of mathematics [1,2]. The characterization of information inequalities has been of interest for decades (see, e.g., [3,4] and references therein), mainly triggered by their indispensable role in proving direct and converse results for channel coding and data compression for single and multi-user information systems. Information inequalities, which apply to classical and generalized information measures, have also demonstrated far-reaching consequences beyond the study of the coding theorems and fundamental limits of communication systems. One such remarkable example (among many) is the usefulness of information measures and information inequalities in providing information–theoretic proofs in the field of combinatorics and graph theory (see, e.g., [5,6,7,8,9,10,11,12,13,14,15,16,17,18,19,20,21,22]).

A basic property that is commonly used for the characterization of information inequalities relies on the nonnegativity of the (conditional and unconditional) Shannon entropy of discrete random variables, the nonnegativity of the (conditional and unconditional) relative entropy and the Shannon mutual information of general random variables, and the chain rules which hold for these classical information measures. A byproduct of these properties is the sub/supermodularity of some classical information measures, which also prove to be useful by taking advantage of the vast literature on sub/supermodular functions and polymatroids [22,23,24,25,26,27,28,29,30,31]. Another instrumental information inequality is the entropy power inequality, which dates back to Shannon [32]. It has been extensively generalized for different types of random variables and generalized entropies, studied in regard to its geometrical relations [33], and it has been also ubiquitously used for the analysis of various information–theoretic problems.

Among the most useful information inequalities are Han’s inequality [34], its generalized versions (e.g., [15,25,30,31]), and Shearer’s lemma [7] with its generalizations and refinements (e.g., [15,31,35]). In spite of their simplicity, these inequalities prove to be useful in information theory, and other diverse fields of mathematics and engineering (see, e.g., [6,35]). More specifically in regard to these inequalities, in Proposition 1 of [22], Madiman and Tetali introduced an information inequality which can be specialized to Han’s inequality, and which also refines Shearer’s lemma while also providing a counterpart result. In [30], Tian generalized Han’s inequality by relying on the sub/supermodularity of the unconditional/conditional Shannon entropy. Likewise, the work in [31] by Kishi et al., relies on the sub/supermodularity properties of Shannon information measures, and it provides refinements of Shearer’s lemma and Han’s inequality. Apart of the refinements of these classical and widely-used inequalities in [31], the suggested approach in the present work can be viewed in a sense as a (nontrivial) generalization and extension of a result in [31] (to be explained in Section 3.2).

This work is focused on the derivation of information inequalities via submodularity and nonnegativity properties, and on a problem in extremal graph theory whose analysis relies on an information inequality. The field of extremal graph theory, which is a subfield of extremal combinatorics, was among the early and fast developing branches of graph theory during the 20th century. Extremal graph theory explores the relations between properties of graphs such as its order, size, chromatic number or maximal and minimal degrees, under some constraints on the graph (by, e.g., considering graphs of a fixed order, and by also imposing a type of a forbidden subgraph). The interested reader is referred to the comprehensive textbooks [10,36] on the vast field of extremal combinatorics and extremal graph theory.

This paper suggests an approach for the derivation of families of inequalities for set functions, and it applies it to obtain information inequalities with Shannon information measures that satisfy sub/supermodularity and monotonicity properties. Some of the derived information inequalities are new, while some known results (such as the generalized version of Han’s inequality [25]) are reproduced as corollaries in a simple and unified way. This paper also applies the generalized Han’s inequality to analyze a problem in extremal graph theory, with an information–theoretic proof and interpretation. The analysis leads to some generalized and refined bounds in comparison to the insightful results in Theorems 4.2 and 4.3 of [6]. For the purpose of the suggested problem and analysis, the presentation here is self-contained.

The paper is structured as follows: Section 2 provides essential notation and preliminary material for this paper. Section 3 presents a new methodology for the derivation of families of inequalities for set functions which satisfy sub/supermodularity properties (Theorem 1). The suggested methodology is then applied in Section 3 for the derivation of information inequalities by relying on sub/supermodularity properties of Shannon information measures. Section 3 also considers connections of the suggested approach to a generalized version of Shearer’s lemma, and to other results in the literature. Most of the results in Section 3 are proved in Section 4. Section 5 applies the generalized Han’s inequality to a problem in extremal graph theory (Theorem 2). A byproduct of Theorem 2, which is of interest in its own right, is also analyzed in Section 5 (Theorem 3). The presentation and analysis in Section 5 is accessible to the reader, independently of the earlier material on information inequalities in Section 3 and Section 4. Some additional proofs, mostly for making the paper self-contained or for suggesting an alternative proof, are relegated to the appendices (Appendix A and Appendix B).

## 2. Preliminaries and Notation

The present section provides essential notation and preliminary material for this paper.

- N≜{1,2,…} denotes the set of natural numbers.
- R denotes the set of real numbers, and $R_{+}$ denotes the set of nonnegative real numbers.
- Ø denotes the empty set.
- $2^{\Omega }≜\left\{A:A\subseteq \Omega \right\}$ denotes the power set of a set Ω (i.e., the set of all subsets of Ω).
- $T^{c}≜\Omega \backslash T$ denotes the complement of a subset T in Ω.
- 1{E} is an indicator of *E*; it is 1 if event *E* is satisfied, and zero otherwise.
- [n]≜{1,…,n} for every n∈N;
- $X^{n}≜\left(X_{1},\ldots ,X_{n}\right)$ denotes an *n*-dimensional random vector;
- $X_{S}≜{\left(X_{i}\right)}_{i\in S}$ is a random vector for a nonempty subset S⊆[n]; if S=Ø, then $X_{S}$ is an empty set, and conditioning on $X_{S}$ is void.
- Let *X* be a discrete random variable that takes its values on a set X, and let PXXYZ be the probability mass function (PMF) of *X*. The *Shannon entropy* of *X* is given by
  (1) H(X)≜−∑x∈XPXXYZ(x)logPXXYZ(x),
  where throughout this paper, we take all logarithms to base 2.
- The *binary entropy function* $H_{b}:\left[0,1\right]\to \left[0,\log2\right]$ is given by
  (2) $$\begin{array}{c} H_{b}\left(p\right)≜-p\log p-\left(1-p\right)\log\left(1-p\right),\;p\in \left[0,1\right], \end{array}$$
  where, by continuous extension, the convention 0log0=0 is used.
- Let *X* and *Y* be discrete random variables with a joint PMF PXYXYZ, and a conditional PMF of *X* given *Y* denoted by PXXYZ|YXYZ. The *conditional entropy* of *X* given *Y* is defined as
  (3a)H(X|Y)≜−∑(x,y)∈X×YPXYXYZ(x,y)logPXXYZ|YXYZ(x|y)(3b)=∑y∈YPYXYZ(y)H(X|Y=y),
  and
  (4) $$\begin{array}{cc} H(X|\;Y) & =H(X,Y)-H(Y). \end{array}$$
- The *mutual information* between *X* and *Y* is symmetric in *X* and *Y*, and it is given by
  (5a)I(X;Y)=H(X)+H(Y)−H(X,Y)(5b)=H(X)−H(X|Y)(5c)=H(Y)−H(Y|X).
- The *conditional mutual information* between two random variables *X* and *Y*, given a third random variable *Z*, is symmetric in *X* and *Y* and it is given by
  (6a)I(X;Y|Z)=H(X|Z)−H(X|Y,Z)(6b)=H(X,Z)+H(Y,Z)−H(Z)−H(X,Y,Z).
- For continuous random variables, the sums in (1) and (3) are replaced with integrals, and the PMFs are replaced with probability densities. The entropy of a continuous random variable is named *differential entropy*.
- For an *n*-dimensional random vector $X^{n}$, the *entropy power* of $X^{n}$ is given by
  (7) $$\begin{array}{c} N\left(X^{n}\right)≜\exp\left(\frac{2}{n}\;H\left(X^{n}\right)\right), \end{array}$$
  where the base of the exponent is identical to the base of the logarithm in (1).

We rely on the following basic properties of the Shannon information measures:

- Conditioning cannot increase the entropy, i.e.,
  (8) $$\begin{array}{c} H(X|\;Y)\leq H(X), \end{array}$$
  with equality in (8) if and only if *X* and *Y* are independent.
- Generalizing (4) to *n*-dimensional random vectors gives the chain rule
  (9) $$\begin{array}{c} H\left(X^{n}\right)=\sum_{i=1}^{n}H\left(X_{i}|\;X^{i-1}\right). \end{array}$$
- The *subadditivity property of the entropy* is implied by (8) and (9):
  (10) $$\begin{array}{c} H\left(X^{n}\right)\leq \sum_{i=1}^{n}H\left(X_{i}\right), \end{array}$$
  with equality in (10) if and only if $X_{1},\ldots ,X_{n}$ are independent random variables.
- *Nonnegativity of the (conditional) mutual information*: In light of (5) and (8), I(X;Y)≥0 with equality if and only if *X* and *Y* are independent. More generally, I(X;Y|Z)≥0 with equality if and only if *X* and *Y* are conditionally independent given *Z*.

Let Ω be a finite and non-empty set, and let $f:2^{\Omega }\to R$ be a real-valued set function (i.e., *f* is defined for all subsets of Ω). The following definitions are used.

**Definition** **1.** (Sub/Supermodular function). *The set function $f:2^{\Omega }\to R$ is* submodular *if*

(11) $$\begin{array}{c} f(T)+f(S)\geq f(T\cup S)+f(T\cap S),\;\forall \;S,T\subseteq \Omega \end{array}$$

*Likewise, f is* supermodular *if −f is submodular.*

An identical characterization of submodularity is the diminishing return property (see, e.g., Proposition 2.2 in [23]), where a set function $f:2^{\Omega }\to R$ is submodular if and only if

(12) $$\begin{array}{c} S\subset T\subset \Omega ,\;\;\omega \in T^{c}\;⟹\;f\left(S\cup \left\{\omega \right\}\right)-f\left(S\right)\geq f\left(T\cup \left\{\omega \right\}\right)-f\left(T\right). \end{array}$$

This means that the larger is the set, the smaller is the increase in *f* when a new element is added.

**Definition** **2.** (Monotonic function). *The set function $f:2^{\Omega }\to R$ is* monotonically increasing *if*

(13) $$\begin{array}{c} S\subseteq T\subseteq \Omega \;⟹\;f(S)\leq f(T). \end{array}$$

*Likewise, f is* monotonically decreasing *if −f is monotonically increasing.*

**Definition** **3.** (Polymatroid, ground set and rank function). *Let $f:2^{\Omega }\to R$ be submodular and monotonically increasing set function with f(Ø)=0. The pair (Ω,f) is called a* polymatroid, Ω *is called a* ground set, *and f is called a* rank function.

**Definition** **4.** (Subadditive function). *The set function $f:2^{\Omega }\to R$ is* subadditive *if, for all S,T⊆Ω,*

(14) $$\begin{array}{c} f(S\cup T)\leq f(S)+f(T). \end{array}$$

A nonnegative and submodular set function is subadditive (this readily follows from (11) and (14)). The next proposition introduces results from [25,28,37]. For the sake of completeness, we provide a proof in Appendix A.

**Proposition** **1.** *Let* Ω *be a finite and non-empty set, and let ${\left\{X_{\omega }\right\}}_{\omega \in \Omega }$ be a collection of discrete random variables. Then, the following holds:*

*(a)*  *The set function $f:2^{\Omega }\to R$, given by*
(15) $$\begin{array}{c} f\left(T\right)≜H\left(X_{T}\right),\;T\subseteq \Omega , \end{array}$$
*is a rank function.*
*(b)*  *The set function $f:2^{\Omega }\to R$, given by*
(16) $$\begin{array}{c} f\left(T\right)≜H\left(X_{T}|\;X_{T^{c}}\right),\;T\subseteq \Omega , \end{array}$$
*is supermodular, monotonically increasing, and f(Ø)=0.*
*(c)*  *The set function $f:2^{\Omega }\to R$, given by*
(17) $$\begin{array}{c} f\left(T\right)≜I\left(X_{T};X_{T^{c}}\right),\;T\subseteq \Omega , \end{array}$$
*is submodular, f(Ø)=0, but f is not a rank function. The latter holds since the equality $f\left(T\right)=f\left(T^{c}\right)$, for all T⊆Ω, implies that f is not a monotonic function.*
*(d)*  *Let U,V⊆Ω be disjoint subsets, and let the entries of the random vector $X_{V}$ be conditionally independent given $X_{U}$. Then, the set function $f:2^{V}\to R$ given by*
(18) $$\begin{array}{c} f\left(T\right)≜I\left(X_{U};X_{T}\right),\;T\subseteq V, \end{array}$$
*is a rank function.*
*(e)*  *Let $X_{\Omega }={\left\{X_{\omega }\right\}}_{\omega \in \Omega }$ be independent random variables, and let $f:2^{\Omega }\to R$ be given by*
(19) $$\begin{array}{c} f\left(T\right)≜H\left(\;\sum_{\omega \in T}X_{\omega }\right),\;T\subseteq \Omega . \end{array}$$
*Then, f is a rank function.*

The following proposition addresses the setting of general alphabets.

**Proposition** **2.** *For general alphabets, the set functions f in (15) and (17)–(19) are submodular, and the set function f in (16) is supermodular with f(Ø)≜0. Moreover, the function in (18) stays to be a rank function, and the function in (19) stays to be monotonically increasing.*

**Proof.**  The sub/supermodularity properties in Proposition 1 are preserved due to the nonnegativity of the (conditional) mutual information. The monotonicity property of the functions in (18) and (19) is preserved also in the general alphabet setting due to (A10) and (A14c), and the mutual information in (18) is nonnegative. □

**Remark** **1.** *In contrast to the entropy of discrete random variables, the differential entropy of continuous random variables is* not *functionally submodular in the sense of Lemma A.2 in [38]. This refers to a different form of submodularity, which was needed by Tao [38] to prove sumset inequalities for the entropy of discrete random variables. A follow-up study in [39] by Kontoyiannis and Madiman required substantially new proof strategies for the derivation of sumset inequalities with the differential entropy of continuous random variables. The basic property which replaces the discrete functional submodularity is the data-processing property of mutual information [39]. In the context of the present work, where the commonly used definition of submodularity is used (see Definition 1), the Shannon entropy of discrete random variables and the differential entropy of continuous random variables are both submodular set functions.*

We rely, in this paper, on the following standard terminology for graphs. An undirected graph *G* is an ordered pair G=(V,E) where V=V(G) is a set of elements, and E=E(G) is a set of 2-element subsets (pairs) of *V*. The elements of *V* are called the vertices of *G*, and the elements of *E* are called the edges of *G*. We use the notation V=V(G) and E=E(G) for the sets of vertices and edges, respectively, in the graph *G*. The number of vertices in a finite graph *G* is called the order of *G*, and the number of edges is called the size of *G*. Throughout this paper, we assume that the graph *G* is undirected and finite; it is also assumed to be a simple graph, i.e., it has no loops (no edge connects a vertex in *G* to itself) and there are no multiple edges which connect a pair of vertices in *G*. If e={u,v}∈E(G), then the vertices *u* and *v* are the two ends of the edge *e*. The elements *u* and *v* are adjacent vertices (neighbors) if they are connected by an edge in *G*, i.e., if e={u,v}∈E(G).

## 3. Inequalities via Submodularity

### 3.1. A New Methodology

The present subsection presents a new methodology for the derivation of families of inequalities for set functions, and in particular inequalities with information measures. The suggested methodology relies, to large extent, on the notion of submodularity of set functions, and it is presented in the next theorem.

**Theorem** **1.** *Let *Ω* be a finite set with |Ω|=n. Let $f:2^{\Omega }\to R$ with f(Ø)=0, and g:R→R. Let the sequence ${\left\{t_{k}^{\left(n\right)}\right\}}_{k=1}^{n}$ be given by*

(20) tk(n)≜1nk∑T⊆Ω:|T|=kgf(T)k,k∈[n].

*(a)*  *If f is submodular, and g is monotonically increasing and convex, then the sequence ${\left\{t_{k}^{\left(n\right)}\right\}}_{k=1}^{n}$ is monotonically decreasing, i.e.,*
(21) $$\begin{array}{c} t_{1}^{\left(n\right)}\geq t_{2}^{\left(n\right)}\geq \ldots \geq t_{n}^{\left(n\right)}=g\left(\frac{f(\Omega )}{n}\right). \end{array}$$
*In particular,*
(22) ∑T⊆Ω:|T|=kgf(T)k≥nkgf(Ω)n,k∈[n].
*(b)*  *If f is submodular, and g is monotonically decreasing and concave, then the sequence ${\left\{t_{k}^{\left(n\right)}\right\}}_{k=1}^{n}$ is monotonically increasing.*
*(c)*  *If f is supermodular, and g is monotonically increasing and concave, then the sequence ${\left\{t_{k}^{\left(n\right)}\right\}}_{k=1}^{n}$ is monotonically increasing.*
*(d)*  *If f is supermodular, and g is monotonically decreasing and convex, then the sequence ${\left\{t_{k}^{\left(n\right)}\right\}}_{k=1}^{n}$ is monotonically decreasing.*

**Proof.**  See Section 4.1. □

**Corollary** **1.** *Let *Ω* be a finite set with |Ω|=n, $f:2^{\Omega }\to R$, and g:R→R be convex and monotonically increasing. If*

- *f is a rank function,*
- *g(0)>0 or there is ℓ∈N such that $g\left(0\right)=\ldots =g^{\left(\ell -1\right)}\left(0\right)=0$ with $g^{\left(\ell \right)}\left(0\right)>0$,*
- *${\left\{k_{n}\right\}}_{n=1}^{\infty }$ is a sequence such that $k_{n}\in \left[n\right]$ for all n∈N with $k_{n}\underset{n\to \infty }{⟶}\infty$,*

*then*

(23) $$\begin{array}{c} \lim_{n\to \infty }\left\{\frac{1}{n}\;\log\left(\sum_{T\subseteq \Omega :\;|T|=k_{n}}g\left(\frac{f(T)}{k_{n}}\right)\right)-H_{b}\left(\frac{k_{n}}{n}\right)\right\}=0, \end{array}$$

*and if $\lim_{n\to \infty }\frac{k_{n}}{n}=\beta \in \left[0,1\right]$, then*

(24) $$\begin{array}{c} \lim_{n\to \infty }\frac{1}{n}\;\log\left(\sum_{T\subseteq \Omega :\;|T|=k_{n}}g\left(\frac{f(T)}{k_{n}}\right)\right)=H_{b}\left(\beta \right). \end{array}$$

**Proof.**  See Section 4.2. □

**Corollary** **2.** *Let *Ω* be a finite set with |Ω|=n, and $f:2^{\Omega }\to R$ be submodular and nonnegative with f(Ø)=0. Then,*

*(a)*  *For α≥1 and k∈[n−1]*
(25) $$\begin{array}{c} \sum_{T\subseteq \Omega :\;|T|=k}\left(f^{\alpha }\left(\Omega \right)-f^{\alpha }\left(T\right)\right)\leq c_{\alpha }\left(n,k\right)\;f^{\alpha }\left(\Omega \right), \end{array}$$
  *with*
(26) cα(n,k)≜1−kαnαnk.
  *For α=1, *(25)* holds with c1(n,k)=n−1k regardless of the nonnegativity of f.*
*(b)*  *If f is also monotonically increasing (i.e., f is a rank function), then for α≥1*
(27) knα−1n−1k−1fα(Ω)≤∑T⊆Ω:|T|=kfα(T)≤nkfα(Ω),k∈[n].

**Proof.**  See Section 4.3. □

Corollary 2 is next specialized to reproduce Han’s inequality [34], and a generalized version of Han’s inequality (Section 4 of [25]).

Let $X^{n}=\left(X_{1},\ldots ,X_{n}\right)$ be a random vector with finite entropies $H(X_{i})$ for all i∈[n]. The set function $f:2^{\left[n\right]}\to \left[0,\infty \right)$, given by $f\left(T\right)=H\left(X_{T}\right)$ for all T⊆[n], is submodular [25] (see Proposition 1a and Proposition 2). From (25), the following holds:

(a) Setting α=1 in (25) implies that, for all k∈[n−1],
  (28a)∑1≤i1<…<ik≤nH(Xn)−H(Xi1,…,Xik)≤1−knnkH(Xn)(28b)=n−1kH(Xn),
(b) Consequently, setting k=n−1 in (28) gives
  (29) $$\begin{array}{c} \sum_{i=1}^{n}\left(H\left(X^{n}\right)-H\left(X_{1},\ldots ,X_{i-1},X_{i+1},\ldots ,X_{n}\right)\right)\leq H\left(X^{n}\right), \end{array}$$
  which gives Han’s inequality.

Further applications of Theorem 1 lead to the next corollary, which partially introduces some known results that have been proved on a case-by-case basis in Theorems 17.6.1–17.6.3 of [1] and Section 2 of [2]. In particular, the monotonicity properties of the sequences in (30) and (32)–(34) were proved in Theorems 1 and 2, and Corollaries 1 and 2 of [2]. Both known and new results are readily obtained here, in a unified way, from Theorem 1. The utility of one of these inequalities in extremal combinatorics is discussed in the continuation to this subsection (see Proposition 3), providing a natural generalization of a beautiful combinatorial result in Section 3.2 of [19].

**Corollary** **3.** *Let ${\left\{X_{i}\right\}}_{i=1}^{n}$ be random variables with finite entropies. Then, the following holds:*

*(a)*  *The sequences*
(30) hk(n)≜1nk∑T⊆[n]:|T|=kH(XT)k,k∈[n],
(31) ℓk(n)≜1nk∑T⊆[n]:|T|=kI(XT;XTc)k,k∈[n]
  *are monotonically decreasing in k. If ${\left\{X_{i}\right\}}_{i=1}^{n}$ are independent, then also the sequence*
(32) mk(n)≜1n−1k−1∑T⊆[n]:|T|=kH∑ω∈TXω,k∈[n]
  *is monotonically decreasing in k.*
*(b)*  *The sequence*
(33) rk(n)≜1nk∑T⊆[n]:|T|=kH(XT|XTc)k,k∈[n]
  *is monotonically increasing in k.*
*(c)*  *For every r>0, the sequences*
(34)sk(n)(r)≜1nk∑T⊆[n]:|T|=kNr(XT),k∈[n],(35)uk(n)(r)≜1nk∑T⊆[n]:|T|=kexp−rH(XT|XTc)k,k∈[n],(36)vk(n)(r)≜1nk∑T⊆[n]:|T|=kexprI(XT;XTc)k,k∈[n]
  *are monotonically decreasing in k. If ${\left\{X_{i}\right\}}_{i=1}^{n}$ are independent, then also the sequence*
(37) wk(n)(r)≜1nk∑T⊆[n]:|T|=kNr∑ω∈TXω,k∈[n]
  *is monotonically decreasing in k.*

**Proof.**  The finite entropies of ${\left\{X_{i}\right\}}_{i=1}^{n}$ assure that the entropies involved in the sequences (30)–(37) are finite. Item (a) follows from Theorem 1a, where the submodular set functions *f* which correspond to (30)–(32) are given in (15), (17) and (19), respectively, and *g* is the identity function on the real line. The identity knk=nn−1k−1 is used for (32). Item (b) follows from Theorem 1c, where *f* is the supermodular function in (16) and *g* is the identity function on the real line. We next prove Item (c). The sequence (34) is monotonically decreasing by Theorem 1a, where *f* is the submodular function in (15), and g:R→R is the monotonically increasing and convex function defined as g(x)=exp(2rx) for x∈R (with r>0). The sequence (35) is monotonically decreasing by Theorem 1d, where *f* is the supermodular function in (16), and g:R→R is the monotonically decreasing and convex function defined as g(x)=exp(−rx) for x∈R. The sequence (36) is monotonically decreasing by Theorem 1a, where *f* is the submodular function in (17) and *g* is the monotonically increasing and convex function defined as g(x)=exp(rx) for x∈R. Finally, the sequence (37) is monotonically decreasing by Theorem 1a, where *f* is the submodular function in (19) and *g* is the monotonically increasing and convex function defined as g(x)=exp(2rx) for x∈R. □

**Remark** **2.** *From Proposition 2, since the proof of Corollary 3 only relies on the sub/supermodularity property of f, the random variables ${\left\{X_{i}\right\}}_{i=1}^{n}$ do not need to be discrete in Corollary 3. In the reproduction of Han’s inequality as an application of Corollary 2, the random variables ${\left\{X_{i}\right\}}_{i=1}^{n}$ do not need to be discrete as well since f is not required to be nonnegative if α=1 (only the submodularity of f in *(15)* is required, which holds due to Proposition 2).*

The following result exemplifies the utility of the monotonicity result of the sequence (30) in extremal combinatorics. It also generalizes the result in Section 3.2 of [19] for an achievable upper bound on the cardinality of a finite set in the three-dimensional Euclidean space, expressed as a function of its number of projections on each of the planes XY,XZ and YZ. The next result provides an achievable upper bound on the cardinality of a finite set of points in an *n*-dimensional Euclidean space, expressed as a function of its number of projections on each of the *k*-dimensional Euclidean subspaces with an arbitrary k<n.

**Proposition** **3.** *Let $P\subseteq R^{n}$ be a finite set of points in the n-dimensional Euclidean space with |P|≜M. Let k∈[n−1], and ℓ≜nk. Let $R_{1},\ldots ,R_{\ell }$ be the projections of P on each of the k-dimensional subspaces of $R^{n}$, and let $|R_{j}|=M_{j}\;$ for all j∈[ℓ]. Then,*

(38) |P|≤∏j=1nkMj1n−1k−1.

*Let $R≜\frac{\log M}{n}$, and $R_{j}≜\frac{\log M_{j}}{k}$ for all j∈[ℓ]. An equivalent form of (38) is given by the inequality*

(39) $$\begin{array}{c} R\leq \frac{1}{\ell }\sum_{j=1}^{\ell }R_{j}. \end{array}$$

*Moreover, if $M_{1}=\ldots =M_{\ell }$ and $\sqrt[{k}]{M_{1}}\in N$, then *(38)* and *(39)* are satisfied with equality if P is a grid of points in $R^{n}$ with $\sqrt[{k}]{M_{1}}$ points on each dimension (so, $M=M_{1}^{\frac{n}{k}}$).*

**Proof.**  Pick uniformly at random a point $X^{n}=\left(X_{1},\ldots ,X_{n}\right)\in P$. Then,

(40) $$\begin{array}{c} H(X^{n})=\log|P|. \end{array}$$

The sequence in (30) is monotonically decreasing, so $h_{k}^{\left(n\right)}\geq h_{n}^{\left(n\right)}$, which is equivalent to

(41) n−1k−1H(Xn)≤∑T⊆[n]:|T|=kH(XT).

Let $S_{1},\ldots ,S_{\ell }$ be the *k*-subsets of the set [n], ordered in a way such that $M_{j}$ is the cardinality of the projection of the set P on the *k*-dimensional subspace whose coordinates are the elements of the subset $S_{j}$. Then, (41) can be expressed in the form

(42) n−1k−1H(Xn)≤∑j=1ℓH(XSj),

and also

(43) $$\begin{array}{c} H\left(X_{S_{j}}\right)\leq \log M_{j},\;j\in \left[\ell \right], \end{array}$$

since the entropy of a random variable is upper bounded by the logarithm of the number of its possible values. Combining (40), (42) and (43) gives

(44) n−1k−1log|P|≤∑j=1ℓlogMj.

Exponentiating both sides of (44) gives (38). In addition, using the identity nk=nkn−1k−1 gives (39) from (44). Finally, the sufficiency condition for equalities in (38) or (39) can be easily verified, which is obtained if P is a grid of points in $R^{n}$ with the same finite number of projections on each dimension. □

### 3.2. Connections to a Generalized Version of Shearer’s Lemma and Other Results in the Literature

The next proposition is a known generalized version of Shearer’s Lemma.

**Proposition** **4.** *Let *Ω* be a finite set, let ${\left\{S_{j}\right\}}_{j=1}^{M}$ be a finite collection of subsets of *Ω* (with M∈N), and let $f:2^{\Omega }\to R$ be a set function.*

*(a)*  *If f is non-negative and submodular, and every element in *Ω* is included in at least d≥1 of the subsets ${\left\{S_{j}\right\}}_{j=1}^{M}$, then*
(45) $$\begin{array}{c} \sum_{j=1}^{M}f\left(S_{j}\right)\geq d\;f\left(\Omega \right). \end{array}$$
*(b)*  *If f is a rank function, A⊂Ω, and every element in A is included in at least d≥1 of the subsets ${\left\{S_{j}\right\}}_{j=1}^{M}$, then*
(46) $$\begin{array}{c} \sum_{j=1}^{M}f\left(S_{j}\right)\geq d\;f\left(A\right). \end{array}$$

The first part of Proposition 4 was pointed out in Section 1.5 of [35], and the second part of Proposition 4 is a generalization of Remark 1 and inequality (47) in [20]. We provide a (somewhat different) proof of Proposition 4a, as well as a self-contained proof of Proposition 4b in Appendix B.

Let ${\left\{X_{i}\right\}}_{i=1}^{n}$ be discrete random variables, and consider the set function $f:2^{\left[n\right]}\to R_{+}$ which is defined as $f\left(A\right)=H\left(X_{A}\right)$ for all A⊆[n]. Since *f* is a rank function [25], Proposition 4 then specializes to Shearer’s Lemma [7] and a modified version of this lemma (see Remark 1 of [20]).

In light of Proposition 1e and Proposition 4b, Corollaries 4 and 5 are obtained as follows.

**Corollary** **4.** *Let ${\left\{X_{i}\right\}}_{i=1}^{n}$ be independent discrete random variables, ${\left\{S_{j}\right\}}_{j=1}^{M}$ be subsets of [n], and A⊆[n]. If each element in A belongs to at least d≥1 of the sets ${\left\{S_{j}\right\}}_{j=1}^{M}$, then*

(47) $$\begin{array}{c} d\;H\left(\;\sum_{i\in A}X_{i}\right)\leq \sum_{j=1}^{M}H\left(\;\sum_{i\in S_{j}}X_{i}\right). \end{array}$$

*In particular, if every i∈[n] is included in at least d≥1 of the subsets ${\left\{S_{j}\right\}}_{j=1}^{M}$, then*

(48) $$\begin{array}{c} d\;H\left(\;\sum_{i=1}^{n}X_{i}\right)\leq \sum_{j=1}^{M}H\left(\;\sum_{i\in S_{j}}X_{i}\right). \end{array}$$

**Remark** **3.** *Inequality *(48)* is also a special case of [37] (Theorem 2), and they coincide if every element i∈[n] is included in a fixed number (d) of the subsets ${\left\{S_{j}\right\}}_{j=1}^{M}$.*

A specialization of Corollary 4 gives the next result.

**Corollary** **5.** *Let ${\left\{X_{i}\right\}}_{i=1}^{n}$ be independent and discrete random variables with finite variances. Then, the following holds:*

*(a)*  *For every k∈[n−1],*
(49) H∑i=1nXi≤1n−1k−1∑T⊆[n]:|T|=kH∑ω∈TXω,
*and equivalently,*
(50) N∑i=1nXi≤∏T⊆[n]:|T|=kN∑ω∈TXω1n−1k−1.
*(b)*  *For every k∈[n−1],*
(51) N∑i=1nXi≤1nk∑T⊆[n]:|T|=kNnk∑ω∈TXω,
  *where *(51)* is in general looser than *(50)*, with equivalence if ${\left\{X_{i}\right\}}_{i=1}^{n}$ are i.i.d.; in particular,*
(52a)N∑i=1nXi≤∏j=1nN∑i≠jXi1n−1(52b)≤1n∑j=1nN∑i≠jXinn−1.

**Proof.**  Let ${\left\{S_{j}\right\}}_{j=1}^{M}$ be all the *k*-element subsets of Ω=[n] (with M=nk). Then, every element i∈[n] belongs to d=kMn=n−1k−1 such subsets, which then gives (49) as a special case of (48). Alternatively, (49) follows from Corollary 3b, which yields $m_{k}^{\left(n\right)}\geq m_{n}^{\left(n\right)}$ for all k∈[n−1]. Exponentiating both sides of (49) gives (50). Inequality (51) is a loosened version of (50), which follows by invoking the AM-GM inequality (i.e., the geometric mean of nonnegative real numbers is less than or equal to their arithmetic mean, with equality between these two means if and only if these numbers are all equal), in conjunction with the identity knnk=n−1k−1. Inequalities (50) and (51) are consequently equivalent if ${\left\{X_{i}\right\}}_{i=1}^{n}$ are i.i.d. random variables, and (52) is a specialized version of (50) and the loosened inequality (51) by setting k=n−1. □

The next remarks consider information inequalities in Corollaries 3–5, in light of Theorem 1 here, and some known results in the literature.

**Remark** **4.** *Inequality *(49)* was derived by Madiman as a special case of Theorem 2 in [37]. The proof of Corollary 5a shows that *(49)* can be also derived in two different ways as special cases of both Theorem 1a and Proposition 4a.*

**Remark** **5.** *Inequality *(51)* can be also derived as a special case of Theorem 1a, where f is the rank function in *(19)*, and g:R→R is given by g(x)≜exp(2nx) for all x∈R. It also follows from the monotonicity property in Corollary 3c, which yields $w_{k}^{\left(n\right)}\left(n\right)\geq w_{n}^{\left(n\right)}\left(n\right)$ for all k∈[n−1].*

**Remark** **6.** *The result in Theorem 8 of [31] is a special case of Theorem 1a here, which follows by taking the function g in Theorem 1a to be the identity function. The flexibility in selecting the function g in Theorem 1 enables to obtain a larger collection of information inequalities. This is in part reflected from a comparison of Corollary 3 here with Corollary 9 of [31]. More specifically, the findings about the monotonicity properties in *(30)*, *(31)* and (*33)* were obtained in Corollary 9 of [31], while relying on Theorem 8 of [31] and the sub/supermodularity properties of the considered Shannon information measures. It is noted, however, that the monotonicity results of the sequences *(34)–(37)* (Corollary 3c) are not implied by Theorem 8 of [31].*

**Remark** **7.** *Inequality *(52)* forms a counterpart of an entropy power inequality by Artstein et al., (Theorem 3 of [40]), where for independent random variables ${\left\{X_{i}\right\}}_{i=1}^{n}$ with finite variances:*

(53) $$\begin{array}{c} N\left(\sum_{i=1}^{n}X_{i}\right)\geq \frac{1}{n-1}\sum_{j=1}^{n}\;N\left(\sum_{i\neq j}X_{i}\right). \end{array}$$

*Inequality *(50)*, and also its looser version in *(51)*, form counterparts of the generalized inequality by Madiman and Barron, which reads (see inequality (4) in [41]):*

(54) N∑i=1nXi≥1n−1k−1∑T⊆[n]:|T|=kN∑ω∈TXω,k∈[n−1].

## 4. Proofs

The present section provides proofs of (most of the) results in Section 3.

### 4.1. Proof of Theorem 1

We prove Item (a), and then readily prove Items (b)–(d). Define the auxiliary sequence

(55) fk(n)≜1nk∑T⊆Ω:|T|=kf(T),k∈[0:n],

averaging *f* over all *k*-element subsets of the *n*-element set $\Omega ≜\{\omega_{1},\ldots ,\omega_{n}\}$. Let the permutation π:[n]→[n] be arbitrary. For k∈[n−1], let

(56a) $$\begin{array}{ccc} & & S_{1}≜\left\{\omega_{\pi (1)},\ldots ,\omega_{\pi (k-1)},\omega_{\pi (k)}\right\}, \end{array}$$

(56b) $$\begin{array}{ccc} & & S_{2}≜\left\{\omega_{\pi (1)},\ldots ,\omega_{\pi (k-1)},\omega_{\pi (k+1)}\right\}, \end{array}$$

which are *k*-element subsets of Ω with k−1 elements in common. Then,

(57) $$\begin{array}{c} f\left(S_{1}\right)+f\left(S_{2}\right)\geq f\left(S_{1}\cup S_{2}\right)+f\left(S_{1}\cap S_{2}\right), \end{array}$$

which holds by the submodularity of *f* (by assumption), i.e.,

(58) $$\begin{array}{cc} \; & f\left(\{\omega_{\pi (1)},\ldots ,\omega_{\pi (k)}\}\right)+f\left(\{\omega_{\pi (1)},\ldots ,\omega_{\pi (k-1)},\omega_{\pi (k+1)}\}\right)\\ \; & \;\geq f\left(\{\omega_{\pi (1)},\ldots ,\omega_{\pi (k+1)}\}\right)+f\left(\{\omega_{\pi (1)},\ldots ,\omega_{\pi (k-1)}\}\right). \end{array}$$

Averaging the terms on both sides of (58) over all the n! permutations π of [n] gives

(59a)1n!∑πf{ωπ(1),…,ωπ(k)}=k!(n−k)!n!∑T⊆Ω:|T|=kf(T)(59b)=1nk∑T⊆Ω:|T|=kf(T)(59c)=fk(n),

and similarly

(60a)1n!∑πf{ωπ(1),…,ωπ(k−1),ωπ(k+1)}=fk(n),(60b)1n!∑πf{ωπ(1),…,ωπ(k+1)}=fk+1(n),(60c)1n!∑πf{ωπ(1),…,ωπ(k−1)}=fk−1(n),

with $f_{0}^{\left(n\right)}=0$ since by assumption f(Ø)=0. Combining (58)–(60) gives

(61) $$\begin{array}{c} 2f_{k}^{\left(n\right)}\geq f_{k+1}^{\left(n\right)}+f_{k-1}^{\left(n\right)},\;k\in \left[n-1\right], \end{array}$$

which is rewritten as

(62) $$\begin{array}{c} f_{k}^{\left(n\right)}-f_{k-1}^{\left(n\right)}\geq f_{k+1}^{\left(n\right)}-f_{k}^{\left(n\right)},\;k\in \left[n-1\right]. \end{array}$$

Consequently, it follows that

(63a)fk(n)k−fk+1(n)k+1=1k∑j=1kfj(n)−fj−1(n)−1k+1∑j=1k+1fj(n)−fj−1(n)(63b)=1k−1k+1∑j=1kfj(n)−fj−1(n)−1k+1fk+1(n)−fk(n)(63c)=1k(k+1)∑j=1kfj(n)−fj−1(n)−fk+1(n)−fk(n)(63d)≥0,

where equality (63a) holds since $f_{0}^{\left(n\right)}=0$, and inequality (63d) holds by (62). The sequence ${\left\{\frac{f_{k}^{\left(n\right)}}{k}\right\}}_{k=1}^{n}$ is therefore monotonically decreasing, and in particular

(64) $$\begin{array}{c} f_{k}^{\left(n\right)}\geq \frac{k\;f_{n}^{\left(n\right)}}{n}=\frac{k}{n}. \end{array}$$

We next prove (25) when α=1, and then proceed to prove Theorem 1. By (64)

(65) $$\begin{array}{c} \frac{f_{n}^{\left(n\right)}}{n}\leq \frac{f_{n-1}^{\left(n\right)}}{n-1}, \end{array}$$

where, by (55),

(66) $$\begin{array}{c} f_{n}^{\left(n\right)}=f\left(\Omega \right),\;f_{n-1}^{\left(n\right)}=\frac{1}{n}\sum_{T\subseteq \Omega :\;|T|=n-1}f\left(T\right). \end{array}$$

Combining (65) and (66) gives

(67) $$\begin{array}{c} \left(n-1\right)\;f\left(\Omega \right)\leq \sum_{T\subseteq \Omega :\;|T|=n-1}f\left(T\right). \end{array}$$

Since there are *n* subsets T⊆Ω with |T|=n−1, rearranging terms in (67) gives (25) for α=1; it is should be noted that, for α=1, the set function *f* does not need to be nonnegative for the satisfiability of (25) (however, this will be required for α>1).

We next prove Item (a). By (20), for k∈[n],

(68a)tk(n)=1nk∑T⊆Ω:|T|=kgf(T)k(68b)=1nk∑T={t1,…,tk}⊆Ωgf{t1,…,tk}k.

Fix $\Omega_{k}≜\left\{t_{1},\ldots ,t_{k}\right\}\subseteq \Omega$, and let $\tilde{f}:2^{\Omega_{k}}\to R$ be the restriction of the function *f* to the subsets of $\Omega_{k}$. Then, $\tilde{f}$ is a submodular set function with $\tilde{f}\left(Ø\right)=0$; similarly to (55), (65) and (66) with *f* replaced by $\tilde{f}$, and *n* replaced by *k*, the sequence ${\left\{\frac{{\tilde{f}}_{j}^{\left(k\right)}}{j}\right\}}_{j=1}^{k}$ is monotonically decreasing. Hence, for k∈[2:n],

(69) $$\begin{array}{c} \frac{{\tilde{f}}_{k}^{\left(k\right)}}{k}\leq \frac{{\tilde{f}}_{k-1}^{\left(k\right)}}{k-1}, \end{array}$$

where

(70a)f˜k(k)=f˜(Ωk)=f{t1,…,tk},(70b)f˜k−1(k)=1k∑T⊆Ωk:|T|=k−1f˜(T)(70c)=1k∑T⊆Ωk:|T|=k−1f(T)(70d)=1k∑i=1kf{t1,…,ti−1,ti+1,…,tk}.

Combining (69) and (70) gives

(71) $$\begin{array}{c} f\left(\{t_{1},\ldots ,t_{k}\}\right)\leq \frac{1}{k-1}\sum_{i=1}^{k}f\left(\{t_{1},\ldots ,t_{i-1},t_{i+1},\ldots ,t_{k}\}\right), \end{array}$$

and, since by assumption *g* is monotonically increasing,

(72) $$\begin{array}{c} g\left(\frac{f\left(\{t_{1},\ldots ,t_{k}\}\right)}{k}\right)\leq g\left(\frac{1}{k}\sum_{i=1}^{k}\frac{f\left(\{t_{1},\ldots ,t_{i-1},t_{i+1},\ldots ,t_{k}\}\right)}{k-1}\right). \end{array}$$

From (68) and (72), for all k∈[2:n],

(73) tk(n)≤1nk∑T={t1,…,tk}⊆Ωg1k∑i=1kf{t1,…,ti−1,ti+1,…,tk}k−1,

and

(74a)tk(n)≤1knk∑i=1k∑T={t1,…,tk}⊆Ωgf{t1,…,ti−1,ti+1,…,tk}k−1(74b)=n−k+1knk∑i=1k∑{t1,…,ti−1,ti+1,…,tk}⊆Ωgf{t1,…,ti−1,ti+1,…,tk}k−1(74c)=k!(n−k)!(n−k+1)n!k∑S⊆Ω:|S|=k−1gf(S)k−1(74d)=(k−1)!(n−k+1)!n!∑S⊆Ω:|S|=k−1gf(S)k−1(74e)=1nk−1∑S⊆Ω:|S|=k−1gf(S)k−1(74f)=tk−1(n),

where (74a) holds by invoking Jensen’s inequality to the convex function *g*; (74b) holds since the term of the inner summation in the right-hand side of (74a) does not depend on $t_{i}$, so for every (k−1)-element subset $S=\{t_{1},\ldots ,t_{i-1},t_{i+1},\ldots ,t_{k}\}\subseteq \Omega$, there are n−k+1 possibilities to extend it by a single element $\left(t_{i}\right)$ into a *k*-element subset $T=\{t_{1},\ldots ,t_{k}\}\subseteq \Omega$; (74e) is straightforward, and (74f) holds by the definition in (20). This proves Item (a).

Item (b) follows from Item (a), and similarly Item (d) follows from Item (c), by replacing *g* with −g. Item (c) is next verified. If *f* is a supermodular set function with f(Ø)=0, then (57) and (58), and (61)–(63) hold with flipped inequality signs. Hence, if *g* is monotonically decreasing, then inequalities (72) and (73) are reversed; finally, if *g* is also concave, then (by Jensen’s inequality) (74) holds with a flipped inequality sign, which proves Item (c).

### 4.2. Proof of Corollary 1

By assumption $f:2^{\Omega }\to R$ is a rank function, which implies that 0≤f(T)≤f(Ω) for every T⊆Ω. Since (by definition) *f* is submodular with f(Ø)=0, and (by assumption) the function *g* is convex and monotonically increasing, then (from (22), while replacing *k* with $k_{n}$)

(75) nkngf(Ω)n≤∑T⊆Ω:|T|=kngf(T)kn≤nkngf(Ω)kn,n∈N.

By the second assumption in Corollary 1, for positive values of *x* that are sufficiently close to zero, we have

- g(x)≈g(0)>0 if g(0)>0;
- g(x) scales like $\frac{1}{\ell !}\;g^{\left(\ell \right)}\left(0\right)\;x^{\ell }$ if $g\left(0\right)=\ldots =g^{\left(\ell -1\right)}\left(0\right)=0$ with $g^{\left(\ell \right)}\left(0\right)>0$ for some ℓ∈N.

In both cases, it follows that

(76) $$\begin{array}{c} \lim_{x\to 0^{+}}x\log g\left(x\right)=0. \end{array}$$

In light of (75) and (76), and since (by assumption) $k_{n}\underset{n\to \infty }{⟶}\infty$, it follows that

(77) limn→∞1nlog∑T⊆Ω:|T|=kngf(T)kn−lognkn=0.

By the following upper and lower bounds on the binomial coefficient:

(78) 1n+1expnHbknn≤nkn≤expnHbknn,

the combination of equalities (77) and (78) gives equality (23). Equality (24) holds as a special case of (23), under the assumption that $\lim_{n\to \infty }\frac{k_{n}}{n}=\beta \in \left[0,1\right]$.

### 4.3. Proof of Corollary 2

For α=1, Corollary 2 is proved in (67). Fix α>1, and let g:R→R be

(79) $$\begin{array}{c} g\left(x\right)≜\{\begin{array}{cc} x^{\alpha }, & x\geq 0,\\ 0, & x<0, \end{array} \end{array}$$

which is monotonically increasing and convex on the real line. By Theorem 1a,

(80) $$\begin{array}{c} t_{k}^{\left(n\right)}\geq t_{n}^{\left(n\right)},\;k\in \left[n\right]. \end{array}$$

Since by assumption *f* is nonnegative, it follows from (20) and (79) that

(81a) tk(n)=1nk∑T⊆Ω:|T|=kgf(T)k

(81b) =1kαnk∑T⊆Ω:|T|=kfα(T).

Combining (80)–(81) and rearranging terms gives, for all α>1,

(82a)∑T⊆Ω:|T|=kfα(T)≥knαnkfα(Ω)(82b)=knα−1n−1k−1fα(Ω),

where equality (82b) holds by the identity knnk=n−1k−1. This further gives

(83a)∑T⊆Ω:|T|=kfα(Ω)−fα(T)=nkfα(Ω)−∑T⊆Ω:|T|=kfα(T)(83b)≤1−kαnαnkfα(Ω)(83c)=cα(n,k)fα(Ω),

where equality (83c) holds by the definition in (26). This proves (25) for α>1.

We next prove Item (b). The function *f* is (by assumption) a rank function, which yields its nonnegativity. Hence, the leftmost inequality in (27) holds by (82). The rightmost inequality in (27) also holds since $f:2^{\Omega }\to R$ is monotonically increasing, which yields f(T)≤f(Ω) for all T⊆Ω. For k∈[n] and α≥0 (in particular, for α≥1),

(84) ∑T⊆Ω:|T|=kfα(T)≤nkfα(Ω),

where (84) holds since there are nk*k*-element subsets T of the *n*-element set Ω, and every summand $f^{\alpha }\left(T\right)$ (with T⊆Ω) is upper bounded by $f^{\alpha }\left(\Omega \right)$.

## 5. A Problem in Extremal Graph Theory

This section applies the generalization of Han’s inequality in (28) to the following problem.

### 5.1. Problem Formulation

Let $A\subseteq {\left\{-1,1\right\}}^{n}$, with n∈N, and let τ∈[n]. Let $G=G_{A,\tau }$ be an un-directed simple graph with vertex set V(G)=A, and pairs of vertices in *G* are adjacent (i.e., connected by an edge) if and only if they are represented by vectors in A whose Hamming distance is less than or equal to τ:

(85) $$\begin{array}{c} \left\{x^{n},y^{n}\right\}\in E\left(G\right)\;\Leftrightarrow \;\left(x^{n},y^{n}\in A,\;\;x^{n}\neq y^{n},\;\;d_{H}\;\left(x^{n},\;y^{n}\right)\leq \tau \right). \end{array}$$

The question is how large can the size of *G* be (i.e., how many edges it may have) as a function of the cardinality of the set A, and possibly based also on some basic properties of the set A?

This problem and its related analysis generalize and refine, in a nontrivial way, the bound in Theorem 4.2 of [6] which applies to the special case where τ=1. The motivation for this extension is next considered.

### 5.2. Problem Motivation

Constraint coding is common in many data recording systems and data communication systems, where some sequences are more prone to error than others, and a constraint on the sequences that are allowed to be recorded or transmitted is imposed in order to reduce the likelihood of error. Given such a constraint, it is then necessary to encode arbitrary user sequences into sequences that obey the constraint.

From an information–theoretic perspective, this problem can be interpreted as follows. Consider a communication channel W:X→Y with input alphabet X and output alphabet Y, and suppose that a constraint is imposed on the sequences that are allowed to be transmitted over the channel. As a result of such a constraint, the information sequences are first encoded into codewords by an error-correction encoder, followed by a constrained encoder that maps these codewords into constrained sequences. Let them be binary *n*-length sequences from the set $A\subseteq {\left\{-1,1\right\}}^{n}$. A channel modulator then modulates these sequences into symbols from X, and the received sequences at the channel output, with alphabet Y, are first demodulated, and then decoded (in a reverse order of the encoding process) by the constrained decoder and error-correction decoder.

Consider a channel model where pairs of binary *n*-length sequences from the set A whose Hamming distance is less than or equal to a fixed number τ share a common output sequence with positive probability, whereas this halts to be the case if the Hamming distance is larger than τ. In other words, we assume that by design, pairs of sequences in A whose Hamming distance is larger than τ cannot be confused in the sense that there does not exist a common output sequence which may be possibly received (with positive probability) at the channel output.

The confusion graph *G* that is associated with this setup is an undirected simple graph whose vertices represent the *n*-length binary sequences in A, and pairs of vertices are adjacent if and only if the Hamming distance between the sequences that they represent is not larger than τ. The size of *G* (i.e., its number of edges) is equal to the number of pairs of sequences in A which may not be distinguishable by the decoder.

Further motivation for studying this problem is considered in the continuation (see Section 5.5).

### 5.3. Analysis

We next derive an upper bound on the size of the graph *G*. Let $X^{n}=\left(X_{1},\ldots ,X_{n}\right)$ be chosen uniformly at random from the set $A\subseteq {\left\{-1,1\right\}}^{n}$, and let PXnXYZ be the PMF of $X^{n}$. Then,

(86) $$\begin{array}{c} P_{X^{n}}\left(x^{n}\right)=\{\begin{array}{cc} \frac{1}{\left|A\right|}, & \;\mathrm{if}\;x^{n}\in A,\\ 0, & \;\mathrm{if}\;x^{n}\notin A, \end{array} \end{array}$$

which implies that

(87) $$\begin{array}{c} H(X^{n})=\log|A|. \end{array}$$

The graph *G* is an un-directed and simple graph with a vertex set V(G)=A (i.e., the vertices of *G* are in one-to-one correspondence with the binary vectors in the set A). Its set of edges E(G) are the edges which connect all pairs of vertices in *G* whose Hamming distance is less than or equal to τ. For d∈[τ], let $E_{d}\left(G\right)$ be the set of edges in *G* which connect all pairs of vertices in *G* whose Hamming distance is equal to *d*, so

(88) $$\left|E\left(G\right)\right|=\sum_{d=1}^{\tau }\left|E_{d}\left(G\right)\right|.$$

For $x^{n}\in {\left\{-1,1\right\}}^{n}$, d∈[n], and integers $k_{1},\ldots ,k_{d}$ such that $1\leq k_{1}<\ldots <k_{d}\leq n$, let

(89) $$\begin{array}{c} {\tilde{x}}^{\left(k_{1},\ldots ,k_{d}\right)}≜\left(x_{1},\ldots ,x_{k_{1}-1},x_{k_{1}+1},\ldots ,x_{k_{d}-1},x_{k_{d}+1},\ldots ,x_{n}\right) \end{array}$$

be a subvector of $x^{n}$ of length n−d, obtained by dropping the bits of $x^{n}$ in positions $k_{1},\ldots ,k_{d}$; if d=n, then $\left(k_{1},\ldots ,k_{n}\right)=\left(1,\ldots ,n\right)$, and ${\tilde{x}}^{\left(k_{1},\ldots ,k_{d}\right)}$ is an empty vector. By the chain rule for the Shannon entropy,

H(Xn)−HX˜(k1,…,kd)(90a)=HXk1,…,Xkd|X˜(k1,…,kd)(90b)=−∑xn∈{−1,1}nPXnXYZ(xn)logPXk1,…,XkdXYZ|X˜(k1,…,kd)XYZxk1,…,xkd|x˜(k1,…,kd)(90c)=−1|A|∑xn∈AlogPXk1,…,XkdXYZ|X˜(k1,…,kd)XYZxk1,…,xkd|x˜(k1,…,kd),

where equality (90c) holds by (86).

For $x^{n}\in {\left\{-1,1\right\}}^{n}$, d∈[n], and integers $k_{1},\ldots ,k_{d}$ such that $1\leq k_{1}<\ldots <k_{d}\leq n$, let

(91) $$\begin{array}{c} {\bar{x}}^{\left(k_{1},\ldots ,k_{d}\right)}≜\left(x_{1},\ldots ,x_{k_{1}-1},-x_{k_{1}},x_{k_{1}+1},\ldots ,x_{k_{d}-1},-x_{k_{d}},x_{k_{d}+1},\ldots ,x_{n}\right), \end{array}$$

where the bits of $x^{n}$ in position $k_{1},\ldots ,k_{d}$ are flipped (in contrast to ${\tilde{x}}^{\left(k_{1},\ldots ,k_{d}\right)}$ where the bits of $x^{n}$ in these positions are dropped), so ${\bar{x}}^{\left(k_{1},\ldots ,k_{d}\right)}\in {\left\{-1,1\right\}}^{n}$ and $d_{H}\;\left(x^{n},\;{\bar{x}}^{\left(k_{1},\ldots ,k_{d}\right)}\right)=d$. Likewise, if $x^{n},y^{n}\in {\left\{-1,1\right\}}^{n}$ satisfy $d_{H}\;\left(x^{n},\;y^{n}\right)=d$, then there exist integers $k_{1},\ldots ,k_{d}$ such that $1\leq k_{1}<\ldots <k_{d}\leq n$ where $y^{n}={\bar{x}}^{\left(k_{1},\ldots ,k_{d}\right)}$ (i.e., the integers $k_{1},\ldots ,k_{d}$ are the positions (in increasing order) where the vectors $x^{n}$ and $y^{n}$ differ).

Let us characterize the set A by its cardinality, and the following two natural numbers:

(a) If $x^{n}\in A$ and ${\bar{x}}^{\left(k_{1},\ldots ,k_{d}\right)}\in A$ for any $\left(k_{1},\ldots ,k_{d}\right)$ such that $1\leq k_{1}<\ldots <k_{d}\leq n$, then there are at least $m_{d}≜m_{d}\left(A\right)$ vectors y∈A whose subvectors ${\tilde{y}}^{\left(k_{1},\ldots ,k_{d}\right)}$ coincide with ${\tilde{x}}^{\left(k_{1},\ldots ,k_{d}\right)}$, i.e., the integer $m_{d}\geq 2$ satisfies
  (92) $$\begin{array}{c} \;m_{d}\leq \min_{\begin{array}{c} x^{n}\in A,\\ 1\leq k_{1}<\ldots <k_{d}\leq n \end{array}}\left|\left\{y^{n}\in A:\;{\tilde{y}}^{\left(k_{1},\ldots ,k_{d}\right)}={\tilde{x}}^{\left(k_{1},\ldots ,k_{d}\right)},\;{\bar{x}}^{\left(k_{1},\ldots ,k_{d}\right)}\in A\right\}\right|. \end{array}$$
  By definition, the integer $m_{d}$ always exists, and
  (93) $$\begin{array}{c} 2\leq m_{d}\leq \min\{2^{d},\;|A|\}. \end{array}$$
  If no information is available about the value of $m_{d}$, then it can be taken by default to be equal to 2 (since by assumption the two vectors $x^{n}\in A$ and $y^{n}≜{\bar{x}}^{\left(k_{1},\ldots ,k_{d}\right)}\in A$ satisfy the equality ${\tilde{y}}^{\left(k_{1},\ldots ,k_{d}\right)}={\tilde{x}}^{\left(k_{1},\ldots ,k_{d}\right)}$).
(b) If $x^{n}\in A$ and ${\bar{x}}^{\left(k_{1},\ldots ,k_{d}\right)}\notin A$ for any $\left(k_{1},\ldots ,k_{d}\right)$ such that $1\leq k_{1}<\ldots <k_{d}\leq n$, then there are at least $\ell_{d}≜\ell_{d}\left(A\right)$ vectors $y^{n}\in A$ whose subvectors ${\tilde{y}}^{\left(k_{1},\ldots ,k_{d}\right)}$ coincide with ${\tilde{x}}^{\left(k_{1},\ldots ,k_{d}\right)}$, i.e., the integer $\ell_{d}\geq 1$ satisfies
  (94) $$\begin{array}{c} \;\ell_{d}\leq \min_{\begin{array}{c} x^{n}\in A,\\ 1\leq k_{1}<\ldots <k_{d}\leq n \end{array}}\left|\left\{y^{n}\in A:\;{\tilde{y}}^{\left(k_{1},\ldots ,k_{d}\right)}={\tilde{x}}^{\left(k_{1},\ldots ,k_{d}\right)},\;{\bar{x}}^{\left(k_{1},\ldots ,k_{d}\right)}\notin A\right\}\right|. \end{array}$$
  By definition, the integer $\ell_{d}$ always exists, and
  (95) $$\begin{array}{c} 1\leq \ell_{d}\leq \min\{2^{d}-1,\;|A|-1\}. \end{array}$$
  Likewise, if no information is available about the value of $\ell_{d}$, then it can be taken by default to be equal to 1 (since $x^{n}\in A$ satisfies the requirement about its subvector ${\tilde{x}}^{\left(k_{1},\ldots ,k_{d}\right)}$ in (94)).

In general, it would be preferable to have the largest possible values of $m_{d}$ and $\ell_{d}$ (i.e., those satisfying inequalities (92) and (94) with equalities, for obtaining a better upper bound on the size of *G* (this point will be clarified in the sequel). If d=1, then $m_{d}=2$ and $\ell_{d}=1$ are the best possible constants (this holds by the definitions in (92) and (94), which can be also verified by the coincidence of the upper and lower bounds in (93) for d=1, as well as those in (95)).

If $x^{n}\in A$, then we distinguish between the following two cases:

- If ${\bar{x}}^{\left(k_{1},\ldots ,k_{d}\right)}\in A$, then
  (96) PXk1,…,XkdXYZ|X˜(k1,…,kd)XYZ(xk1,…,xkd|x˜(k1,…,kd))≤1md,
  which holds by the way that $m_{d}$ is defined in (92), and since $X^{n}$ is randomly selected to be equiprobable in the set A.
- If ${\bar{x}}^{\left(k_{1},\ldots ,k_{d}\right)}\notin A$, then
  (97) PXk1,…,XkdXYZ|X˜(k1,…,kd)XYZ(xk1,…,xkd|x˜(k1,…,kd))≤1ℓd,
  which holds by the way that $\ell_{d}$ is defined in (94), and since $X^{n}$ is equiprobable on A.

For d∈[τ] and $1\leq k_{1}<\ldots <k_{d}\leq n$, it follows from (90), (96) and (97) that

(98) $$\begin{array}{cc} H\left(X^{n}\right)-H\left({\tilde{X}}^{\left(k_{1},\ldots ,k_{d}\right)}\right) & \geq \frac{\log m_{d}}{\left|A\right|}\;\sum_{x^{n}}1\left\{x^{n}\in A,\;{\bar{x}}^{\left(k_{1},\ldots ,k_{d}\right)}\in A\right\}\\ \; & \;+\frac{\log\ell_{d}}{\left|A\right|}\;\sum_{x^{n}}1\left\{x^{n}\in A,\;{\bar{x}}^{\left(k_{1},\ldots ,k_{d}\right)}\notin A\right\}, \end{array}$$

which, by summing on both sides of inequality (98) over all integers $k_{1},\ldots ,k_{d}$ such that $1\leq k_{1}<\ldots <k_{d}\leq n$, yields

(99) $$\begin{array}{cc} \; & \;\sum_{\begin{array}{c} (k_{1},\ldots ,k_{d}):\\ 1\leq k_{1}<\ldots <k_{d}\leq n \end{array}}\left(H\left(X^{n}\right)-H\left({\tilde{X}}^{\left(k_{1},\ldots ,k_{d}\right)}\right)\right)\\ \; & \geq \frac{\log m_{d}}{\left|A\right|}\;\sum_{\begin{array}{c} (k_{1},\ldots ,k_{d}):\\ 1\leq k_{1}<\ldots <k_{d}\leq n \end{array}}\sum_{x^{n}}1\left\{x^{n}\in A,\;{\bar{x}}^{\left(k_{1},\ldots ,k_{d}\right)}\in A\right\}\\ \; & \;+\frac{\log\ell_{d}}{\left|A\right|}\;\sum_{\begin{array}{c} (k_{1},\ldots ,k_{d}):\\ 1\leq k_{1}<\ldots <k_{d}\leq n \end{array}}\sum_{x^{n}}1\left\{x^{n}\in A,\;{\bar{x}}^{\left(k_{1},\ldots ,k_{d}\right)}\notin A\right\}. \end{array}$$

Equality holds in (99) if the minima on the RHS of (92) and (94) are attained by any element in these sets, and if (92) and (94) are satisfied with equalities (i.e., $m_{d}$ and $\ell_{d}$ are the maximal integers to satisfy inequalities (92) and (94) for the given set A). Hence, this equality holds in particular for d=1, with the constants $m_{d}=2$ and $\ell_{d}=1$.

The double sum in the first term on the RHS of (99) is equal to

(100) $$\begin{array}{c} \sum_{\begin{array}{c} (k_{1},\ldots ,k_{d}):\\ 1\leq k_{1}<\ldots <k_{d}\leq n \end{array}}\sum_{x^{n}}1\left\{x^{n}\in A,\;{\bar{x}}^{\left(k_{1},\ldots ,k_{d}\right)}\in A\right\}=2\;\left|E_{d}\left(G\right)\right|, \end{array}$$

since every pair of adjacent vertices in G that refer to vectors in A whose Hamming distance is equal to *d* is of the form $x^{n}\in A$ and ${\bar{x}}^{\left(k_{1},\ldots ,k_{d}\right)}\in A$, and vice versa, and every edge $\left\{x^{n},{\bar{x}}^{\left(k_{1},\ldots ,k_{d}\right)}\right\}\in E_{d}\left(G\right)$ is counted twice in the double summation on the LHS of (100). For calculating the double sum in the second term on the RHS of (99), we first calculate the sum of these two double summations:

$$\begin{array}{ccc} & & \;\sum_{\begin{array}{c} (k_{1},\ldots ,k_{d}):\\ 1\leq k_{1}<\ldots <k_{d}\leq n \end{array}}\sum_{x^{n}}1\left\{x^{n}\in A,\;{\bar{x}}^{\left(k_{1},\ldots ,k_{d}\right)}\in A\right\}+\sum_{\begin{array}{c} (k_{1},\ldots ,k_{d}):\\ 1\leq k_{1}<\ldots <k_{d}\leq n \end{array}}\sum_{x^{n}}1\left\{x^{n}\in A,\;{\bar{x}}^{\left(k_{1},\ldots ,k_{d}\right)}\notin A\right\} \end{array}$$

(101a)=∑(k1,…,kd):1≤k1<…<kd≤n∑xn1{xn∈A,x¯(k1,…,kd)∈A}+1{xn∈A,x¯(k1,…,kd)∉A}(101b)=∑(k1,…,kd):1≤k1<…<kd≤n∑xn1{xn∈A}(101c)=∑(k1,…,kd):1≤k1<…<kd≤n|A|(101d)=nd|A|,

so, subtracting (100) from (101d) gives that

(102) ∑(k1,…,kd):1≤k1<…<kd≤n∑xn1{xn∈A,x¯(k1,…,kd)∉A}=nd|A|−2Ed(G).

Substituting (100) and (102) into the RHS of (99) gives that, for all d∈[τ],

∑(k1,…,kd):1≤k1<…<kd≤nH(Xn)−HX˜(k1,…,kd)(103a)≥2|Ed(G)|logmd|A|+logℓd|A|nd|A|−2Ed(G)(103b)=ndlogℓd+2|Ed(G)||A|logmdℓd,

with the same necessary and sufficient condition for equality in (103a) as in (99). (Recall that it is in particular an equality for d=1, where in this case $m_{1}=2$ and $\ell_{1}=1$.)

By the generalized Han’s inequality in (28),

(104a)∑(k1,…,kd):1≤k1<…<kd≤nH(Xn)−HX˜(k1,…,kd)≤n−1d−1H(Xn)(104b)=n−1d−1log|A|,

where equality (104b) holds by (87). Combining (103) and (104) yields

(105) n−1d−1log|A|≥ndlogℓd+2|Ed(G)||A|logmdℓd,

and, by the identity nd=ndn−1d−1, we get

(106) |Ed(G)|≤n−1d−1|A|log|A|−ndlogℓd2logmdℓd.

This upper bound is specialized, for d=1, to Theorem 4.2 of [6] (where, by definition, $m_{1}=2$ and $\ell_{1}=1$). This gives that the number of edges in *G*, connecting pairs of vertices which refer to binary vectors in A whose Hamming distance is 1 from each other, satisfies

(107) $$\begin{array}{c} |E_{1}\left(G\right)|\leq \frac{1}{2}\;|A|\;\log_{2}\left|A\right|. \end{array}$$

It is possible to select, by default, the values of the integers $m_{d}$ and $\ell_{d}$ to be equal to 2 and 1, respectively, independently of the value of d∈[τ]. It therefore follows that the upper bound in (106) can be loosened to

(108) |Ed(G)|≤12n−1d−1|A|log2|A|.

This shows that the bound in (108) generalizes the result in Theorem 4.2 of [6], based only on the knowledge of the cardinality of A. Furthermore, the bound (108) can be tightened by the refined bound (106) if the characterization of the set A allows one to assert values for $m_{d}$ and $\ell_{d}$ that are larger than the trivial values of 2 and 1, respectively.

In light of (88) and (108), the number of edges in the graph *G* satisfies

(109) |E(G)|≤12∑d=1τn−1d−1|A|log2|A|,

and if $\tau \leq \frac{n+1}{2}$, then it follows that

(110) $$\begin{array}{c} \left|E\left(G\right)\right|\leq \frac{1}{2}\exp\left(\left(n-1\right)\;H_{b}\left(\frac{\tau -1}{n-1}\right)\right)\;|A|\;\log_{2}\left|A\right|. \end{array}$$

Indeed, the transition from (109) to (110) holds by the inequality

(111) ∑k=0nθnk≤expnHb(θ),θ∈0,12,

where the latter bound is asymptotically tight in the exponent of *n* (for sufficiently large values of *n*).

### 5.4. Comparison of Bounds

We next consider the tightness of the refined bound (106) and the loosened bound (108). Since A is a subset of the *n*-dimensional cube ${\left\{-1,1\right\}}^{n}$, every point in A has at most nd neighbors in A with Hamming distance *d*, so

(112) |Ed(G)|≤12nd|A|.

Comparing the bound on the RHS of (106) with the trivial bound in (112) shows that the former bound is useful if and only if

(113) $$\begin{array}{c} \frac{\log|A|-\frac{n}{d}\;\log\ell_{d}}{\log\frac{m_{d}}{\ell_{d}}}\leq \frac{n}{d}, \end{array}$$

which is obtained by relying on the identity nd=ndn−1d−1. Rearranging terms in (113) gives the necessary and sufficient condition

(114) $$\begin{array}{c} \left|A\right|\leq {\left(m_{d}\right)}^{\frac{n}{d}}, \end{array}$$

which is independent of the value of $\ell_{d}$. Since, by definition, $m_{d}\geq 2$, inequality (114) is automatically satisfied if the stronger condition

(115) $$\begin{array}{c} \left|A\right|\leq 2^{\frac{n}{d}} \end{array}$$

is imposed. The latter also forms a necessary and sufficient condition for the usefulness of the looser bound on the RHS of (108) in comparison to (112).

**Example** **1.** *Suppose that the set $A\subseteq {\left\{-1,1\right\}}^{n}$ is characterized by the property that for all d∈[τ], with a fixed integer τ∈[n], if $x^{n}\in A$ and ${\bar{x}}^{\left(k_{1},\ldots ,k_{d}\right)}\in A$ then all vectors $y^{n}\in {\left\{-1,1\right\}}^{n}$ which coincide with $x^{n}$ and ${\bar{x}}^{\left(k_{1},\ldots ,k_{d}\right)}$ in their (n−d) agreed positions are also included in the set A. Then, for all d∈[τ], we get by definition that $m_{d}=2^{d}$, which yields $\tau \leq \lfloor \log_{2}|A|\rfloor$. Setting $m_{d}=2^{d}$ and the default value $\ell_{d}=1$ on the RHS of *(106)* gives*

(116a)|Ed(G)|≤n−1d−1|A|log|A|−ndlogℓd2logmdℓd(116b)=n−1d−1|A|log|A|2log(2d)(116c)=12dn−1d−1|A|log2|A|(116d)=12nd|A|·log2|A|n.

*Unless $A={\left\{-1,1\right\}}^{n}$, the upper bound on the RHS of *(116d)* is strictly smaller than the trivial upper bound on the RHS of *(112)*. This improvement is consistent with the satisfiability of the (necessary and sufficient) condition in *(115)*, which is strictly satisfied since*

(117) $$\begin{array}{c} |A|<2^{n}={\left(2^{d}\right)}^{\frac{n}{d}}={\left(m_{d}\right)}^{\frac{n}{d}}. \end{array}$$

*On the other hand, the looser upper bound on the RHS of *(108)* gives*

(118) |Ed(G)|≤12nd|A|·dlog2|A|n,

*which is d times larger than the refined bound on the RHS of *(116d)* (since it is based on the exact value of $m_{d}$ for the set A, rather than taking the default value of 2), and it is worse than the trivial bound if and only if $|A|>2^{\frac{n}{d}}$. The latter finding is consistent with *(115)*.*

*This exemplifies the utility of the refined upper bound on the RHS of *(106)* in comparison to the bound on the RHS of *(108)*, where the latter generalizes Theorem 4.2 of [6] from the case where d=1 to all d∈[n]. As it is explained above, this refinement is irrelevant in the special case where d=1, though it proves to be useful in general for d∈[2:n] (as it is exemplified here).*

The following theorem introduces the results of our analysis (so far) in the present section.

**Theorem** **2.** *Let $A\subseteq {\left\{-1,1\right\}}^{n}$, with n∈N, and let τ∈[n]. Let G=(V(G),E(G)) be an un-directed, simple graph with vertex set V(G)=A, and edges connecting pairs of vertices in G which are represented by vectors in A whose Hamming distance is less than or equal to τ. For d∈[τ], let $E_{d}\left(G\right)$ be the set of edges in G which connect all pairs of vertices that are represented by vectors in A whose Hamming distance is equal to d (i.e., $\left|E\left(G\right)\right|=\sum_{d=1}^{\tau }\left|E_{d}\left(G\right)\right|$).*

*(a)*  *For d∈[τ], let the integers $m_{d}\in [2:\min\{2^{d},\;|A|\}]$ and $\ell_{d}\in [\min\{2^{d}-1,\;|A|-1\}]$ (be, preferably, the maximal possible values to) satisfy the requirements in *(92)* and *(94)*, respectively. Then,*
(119) |Ed(G)|≤n−1d−1|A|log|A|−ndlogℓd2logmdℓd.
*(b)*  *A loosened bound, which only depends on the cardinality of the set A, is obtained by setting the default values $m_{d}=2$ and $\ell_{d}=1$. It is then given by*
(120) |Ed(G)|≤12n−1d−1|A|log2|A|,d∈[τ],
*and, if $\tau \leq \frac{n+1}{2}$, then the (overall) number of edges in G satisfies*
(121) $$\begin{array}{c} \left|E\left(G\right)\right|\leq \frac{1}{2}\exp\left(\left(n-1\right)\;H_{b}\left(\frac{\tau -1}{n-1}\right)\right)\;|A|\;\log_{2}\left|A\right|. \end{array}$$
*(c)*  *The refined upper bound on the RHS of *(119)* and the loosened upper bound on the RHS of *(120)* improve the trivial bound 12nd|A|, if and only if $|A|<{\left(m_{d}\right)}^{\frac{n}{d}}$ or $|A|<2^{\frac{n}{d}}$, respectively (see Example 1).*

### 5.5. Influence of Fixed-Size Subsets of Bits

The result in Theorem 4.2 of [6], which is generalized and refined in Theorem 2 here, is turned to study the total influence of the *n* variables of an equiprobable random vector $X^{n}\in {\left\{-1,1\right\}}^{n}$ on a subset $A\subset {\left\{-1,1\right\}}^{n}$. To this end, let ${\bar{X}}^{\left(i\right)}$ denote the vector where the bit at the *i*-th position of $X^{n}$ is flipped, so ${\bar{X}}^{\left(i\right)}≜\left(X_{1},\ldots ,X_{i-1},-X_{i},X_{i+1},\ldots ,X_{n}\right)$ for all i∈[n]. Then, the influence of the *i*-th variable is defined as

(122) $$\begin{array}{c} I_{i}\left(A\right)≜\mathrm{Pr}\left[1\left\{X^{n}\in A\right\}\neq 1\left\{{\bar{X}}^{\left(i\right)}\in A\right\}\right],\;i\in \left[n\right], \end{array}$$

and their total influence is defined to be the sum

(123) $$\begin{array}{c} I\left(A\right)≜\sum_{i=1}^{n}I_{i}\left(A\right). \end{array}$$

As it is shown in Chapters 9 and 10 of [6], influences of subsets of the binary hypercube have far reaching consequences in the study of threshold phenomena, and many other areas. As a corollary of (107), it is obtained in Theorem 4.3 of [6] that, for every subset $A\subset {\left\{-1,1\right\}}^{n}$,

(124) $$\begin{array}{c} I\left(A\right)\geq 2\mathrm{Pr}\left(A\right)\;\log_{2}\frac{1}{\mathrm{Pr}(A)}, \end{array}$$

where $\mathrm{Pr}\left(A\right)≜P\left[X^{n}\in A\right]=\frac{\left|A\right|}{2^{n}}$ by the equiprobable distribution of $X^{n}$ over ${\left\{-1,1\right\}}^{n}$.

In light of Theorem 2, the same approach which is used in Section 4.4 of [6] for the transition from (107) to (124) can be also used to obtain, as a corollary, a lower bound on the average total influence over all subsets of *d* variables. To this end, let $k_{1},\ldots ,k_{d}$ be integers such that $1\leq k_{1}<\ldots <k_{d}\leq n$, and the influence of the variables in positions $k_{1},\ldots ,k_{d}$ be given by

(125) $$\begin{array}{c} I_{\left(k_{1},\ldots ,k_{d}\right)}\left(A\right)≜\mathrm{Pr}\left[1\left\{X^{n}\in A\right\}\neq 1\left\{{\bar{X}}^{\left(k_{1},\ldots ,k_{d}\right)}\in A\right\}\right]. \end{array}$$

Then, let the average influence of subsets of *d* variables be defined as

(126) I(n,d)(A)≜1nd∑(k1,…,kd):1≤k1<…<kd≤nI(k1,…,kd)(A).

Hence, by (123) and (126), $I^{\left(n,1\right)}\left(A\right)=\frac{1}{n}\;I\left(A\right)$ for every subset $A\subset {\left\{-1,1\right\}}^{n}$. Let

(127) $$\begin{array}{c} B^{\left(n,d\right)}\left(A\right)≜\left\{\left(x^{n},y^{n}\right):\;x^{n}\in A,\;y^{n}\in {\left\{-1,1\right\}}^{n}\backslash A,\;d_{H}\;\left(x^{n},\;y^{n}\right)=d\right\}, \end{array}$$

be the set of ordered pairs of sequences $\left(x^{n},y^{n}\right)$, where $x^{n},y^{n}\in {\left\{-1,1\right\}}^{n}$ are of Hamming distance *d* from each other, with $x^{n}\in A$ and $y^{n}\notin A$. By the equiprobable distribution of $X^{n}$ on ${\left\{-1,1\right\}}^{n}$, we get

(128a)I(n,d)(A)=1nd∑(k1,…,kd):1≤k1<…<kd≤nPr1{Xn∈A}≠1X¯(k1,…,kd)∈A(128b)=2nd∑(k1,…,kd):1≤k1<…<kd≤nPrXn∈A,X¯(k1,…,kd)∉A(128c)=2nd·|B(n,d)(A)|2n(128d)=|B(n,d)(A)|2n−1nd.

Since every point in A has nd neighbors of Hamming distance *d* in the set ${\left\{-1,1\right\}}^{n}$, it follows that

(129) nd|A|=2Ed(G)+B(n,d)(A),

where *G* is introduced in Theorem 2, and $E_{d}\left(G\right)$ is the set of edges connecting pairs of vertices in *G* which are represented by vectors in A of Hamming distance *d*. The multiplication by 2 on the RHS of (129) is because every edge whose two endpoints are in the set A is counted twice. Hence, by (106) and (129),

(130a)B(n,d)(A)=nd|A|−2Ed(G)(130b)≥nd|A|−n−1d−1|A|log|A|−ndlogℓdlogmdℓd(130c)=nd|A|1−dnlog|A|−logℓdlogmdℓd(130d)=nd|A|logmd−dnlog|A|logmdℓd,

and the lower bound on the RHS of (130d) is positive if and only if $|A|<{\left(m_{d}\right)}^{\frac{n}{d}}$ (see also (114)). This gives from (128) that the average influence of subsets of *d* variables satisfies

(131a)I(n,d)(A)≥|A|2n−1logmd−dnlog|A|logmdℓd(131b)=2Pr(A)logmd−dnlog2nPr(A)logmdℓd(131c)=2Pr(A)dnlog1Pr(A)−log2dmdlogmdℓd.

Note that by setting d=1, and the default values $m_{d}=2$ and $\ell_{d}=1$ on the RHS of (131c) gives the total influence of the *n* variables satisfies, for all $A\subseteq {\left\{-1,1\right\}}^{n}$,

(132a)I(A)=nI(n,1)(A)(132b)≥2Pr(A)log21Pr(A),

which is then specialized to the result in (Theorem 4.3 of [6], see (124)). This gives the following result.

**Theorem** **3.** *Let $X^{n}$ be an equiprobable random vector over the set ${\left\{-1,1\right\}}^{n}$, let d∈[n] and $A\subset {\left\{-1,1\right\}}^{n}$. Then, the average influence of subsets of d variables of $X^{n}$, as it is defined in (126), is lower bounded as follows:*

(133) $$\begin{array}{cc} I^{\left(n,d\right)}\left(A\right) & \geq 2\mathrm{Pr}\left(A\right)\;\left(\frac{\frac{d}{n}\;\log\frac{1}{\mathrm{Pr}(A)}-\log\frac{2^{d}}{m_{d}}}{\log\frac{m_{d}}{\ell_{d}}}\right), \end{array}$$

*where $\mathrm{Pr}\left(A\right)≜P\left[X^{n}\in A\right]=\frac{\left|A\right|}{2^{n}}$, and the integers $m_{d}$ and $\ell_{d}$ are introduced in Theorem 2. Similarly to the refined upper bound in Theorem 2, the lower bound on the RHS of (133) is informative (i.e., positive) if and only if $|A|<{\left(m_{d}\right)}^{\frac{n}{d}}$. The lower bound on the RHS of (133) can be loosened (by setting the default values $m_{d}=2$ and $\ell_{d}=1$) to*

(134) $$\begin{array}{cc} I^{\left(n,d\right)}\left(A\right) & \geq 2\mathrm{Pr}\left(A\right)\;\left(\frac{d}{n}\;\log_{2}\frac{1}{\mathrm{Pr}(A)}+1-d\right). \end{array}$$

## Data Availability

Not applicable.

## Appendix A. Proof of Proposition 1

For completeness, we prove Proposition 1 which introduces results from [25,28,37].

Let Ω be a non-empty finite set, and let ${\left\{X_{\omega }\right\}}_{\omega \in \Omega }$ be a collection of discrete random variables. We first prove Item (a), showing that the entropy set function $f:2^{\Omega }\to R$ in (15) is a rank function.

- f(Ø)=0.
- Submodularity: If S,T⊆Ω, then
  f(T∪S)+f(T∩S)(A1a)=H(XT∪S)+H(XT∩S)(A1b)=H(XT\S,XT∩S,XS\T)+H(XT∩S)(A1c)=H(XT\S,XS\T|XT∩S)+2H(XT∩S)=H(XT\S|XT∩S)+H(XS\T|XT∩S)−I(XT\S;XS\T|XT∩S)(A1d)+2H(XT∩S)=H(XT\S|XT∩S)+H(XT∩S)+H(XS\T|XT∩S)+H(XT∩S)(A1e)−I(XT\S;XS\T|XT∩S)(A1f)=H(XT\S,XT∩S)+H(XS\T,XT∩S)−I(XT\S;XS\T|XT∩S)(A1g)=H(XT)+H(XS)−I(XT\S;XS\T|XT∩S)(A1h)=f(T)+f(S)−I(XT\S;XS\T|XT∩S),
  which gives
  (A2) $$\begin{array}{c} \;f\left(T\right)+f\left(S\right)-\left[f(T\cup S)+f(T\cap S)\right]=I\left(X_{T\backslash S};X_{S\backslash T}\;|\;X_{T\cap S}\right)\geq 0. \end{array}$$
  This proves the submodularity of *f*, while also showing that
  (A3) $$\begin{array}{c} \;f\left(T\right)+f\left(S\right)=f\left(T\cup S\right)+f\left(T\cap S\right)\;⟺\;X_{T\backslash S}⊥\;\;\;⊥X_{S\backslash T}\;|\;X_{T\cap S}\;, \end{array}$$
  i.e., the rightmost side of (A2) holds with equality if and only if $X_{T\backslash S}$ and $X_{S\backslash T}$ are conditionally independent given $X_{T\cap S}$.
- Monotonicity: If S⊆T⊆Ω, then
  (A4a)f(S)=H(XS)(A4b)≤H(XS)+H(XT|XS)(A4c)=H(XT)(A4d)=f(T),
  so *f* is monotonically increasing.

We next prove Item (b). Consider the set function *f* in (16).

- f(Ø)=0, and $f\left(\Omega \right)=H\left(X_{\Omega }\right)$.
- Supermodularity: If S,T⊆Ω, then
  (A5a)f(T∪S)+f(T∩S)=H(XT∪S|XTc∩Sc)+H(XT∩S|XTc∪Sc)(A5b)=H(XΩ)−H(XTc∩Sc)+H(XΩ)−H(XTc∪Sc)(A5c)=2H(XΩ)−H(XTc∪Sc)+H(XTc∩Sc)(A5d)≥2H(XΩ)−H(XTc)+H(XSc)(A5e)=H(XΩ)−H(XTc)+H(XΩ)−H(XSc)(A5f)=H(XT|XTc)+H(XS|XSc)(A5g)=f(T)+f(S),
  where inequality (A5d) holds since the entropy function in (15) is submodular (by Item (a)).
- Monotonicity: If S⊆T⊆Ω, then
  (A6a)f(S)=H(XS|XSc)(A6b)≤H(XS|XTc)(Tc⊆Sc)(A6c)≤H(XT|XTc)(A6d)=f(T),
  so *f* is monotonically increasing.

Item (c) follows easily from Items (a) and (b). Consider the set function $f:2^{\Omega }\to R$ in (17). Then, for all T∈Ω, $f\left(T\right)=I\left(X_{T};X_{T^{c}}\right)=H\left(X_{T}\right)-H\left(X_{T}|\;X_{T^{c}}\right)$, so *f* is expressed as a difference of a submodular function and a supermodular function, which gives a submodular function. Furthermore, f(Ø)=0; by the symmetry of the mutual information, $f\left(T\right)=f\left(T^{c}\right)$ for all T⊆Ω, so *f* is not monotonic.

We next prove Item (d). Consider the set function $f:2^{V}\to R$ in (18), and we need to prove that *f* is submodular under the conditions in Item (d) where U,V⊆Ω are disjoint subsets, and the entries of the random vector $X_{V}$ are conditionally independent given $X_{U}$.

- $f\left(Ø\right)=I\left(X_{U};X_{Ø}\right)=0$.
- Submodularity: If S,T⊆V, then
  f(T∪S)+f(T∩S)(A7a)=I(XU;XT∪S)+I(XU;XT∩S)(A7b)=H(XT∪S)−H(XT∪S|XU)+H(XT∩S)−H(XT∩S|XU)(A7c)=H(XT∪S)+H(XT∩S)−H(XT∪S|XU)+H(XT∩S|XU)=H(XT)+H(XS)−I(XT\S;XS\T|XT∩S)(A7d)−H(XT∪S|XU)+H(XT∩S|XU),
  where equality (A7d) holds by the proof of Item (a) (see (A2)). By the assumption on the conditional independence of the random variables ${\left\{X_{v}\right\}}_{v\in V}$ given $X_{U}$, we get
  (A8a)H(XT∪S|XU)+H(XT∩S|XU)=∑ω∈T∪SH(Xω|XU)+∑ω∈T∩SH(Xω|XU)(A8c)=∑ω∈TH(Xω|XU)+∑ω∈SH(Xω|XU)(A8c)=H(XT|XU)+H(XS|XU).
  Consequently, combining (A7) and (A8) gives
  f(T∪S)+f(T∩S)=H(XT)+H(XS)−I(XT\S;XS\T|XT∩S)(A9a)−H(XT|XU)+H(XS|XU)=H(XT)−H(XT|XU)+H(XS)−H(XS|XU)(A9b)−I(XT\S;XS\T|XT∩S)(A9c)=I(XT;XU)+I(XS;XU)−I(XT\S;XS\T|XT∩S)(A9d)=f(T)+f(S)−I(XT\S;XS\T|XT∩S)(A9e)≤f(T)+f(S),
  where the inequality (A9e) holds with equality if and only if $X_{T\backslash S}$ and $X_{S\backslash T}$ are conditionally independent given $X_{T\cap S}$.
- Monotonicity: If S⊆T⊆V, then
  (A10) $$\begin{array}{c} f\left(S\right)=I\left(X_{U};X_{S}\right)\leq I\left(X_{U};X_{T}\right)=f\left(T\right), \end{array}$$
  so *f* is monotonically increasing.

We finally prove Item (e), where it is needed to show that the entropy of a sum of independent random variables is a rank function. Let $f:2^{\Omega }\to R$ be the set function as given in (19).

- f(Ø)=0.
- Submodularity: Let S,T⊆Ω. Define
  (A11) $$\begin{array}{c} U≜\sum_{\omega \in T\cap S}X_{\omega },\;V≜\sum_{\omega \in S\backslash T}X_{\omega },\;W≜\sum_{\omega \in T\backslash S}X_{\omega }. \end{array}$$
  From the independence of the random variables ${\left\{X_{\omega }\right\}}_{\omega \in \Omega }$, it follows that U,V and *W* are independent. Hence, we get
  f(T)+f(S)−f(T∪S)+f(T∩S)(A12a)=f(T)−f(T∩S)−f(T∪S)−f(S)(A12b)=H(U+W)−H(U)−H(U+V+W)−H(U+V)(A12c)=H(U+W)−H(U+W|W)−H(U+V+W)−H(U+V)(A12d)=H(U+W)−H(U+W|W)−H(U+V+W)−H(U+V+W|W)(A12e)=I(U+W;W)−I(U+V+W;W)(A12f)≥I(U+W;W)−I(U+W,V;W),
  and
  (A13a)I(U+W,V;W)=I(V;W)+I(U+W;W|V)(A13b)=I(U+W;W|V)(A13c)=I(U+W;W).
  Combining (A12) and (A13) gives (11).
- Monotonicity: If S⊆T⊆Ω, then since ${\left\{X_{\omega }\right\}}_{\omega \in \Omega }$ are independent random variables, (A11) implies that *U* and *W* are independent and V=0. Hence,
  (A14a)f(T)−f(S)=H(U+W)−H(U)(A14b)=H(U+W)−H(U+W|W)(A14c)=I(U+W;W)≥0.

This completes the proof of Proposition 1.

## Appendix B. Proof of Proposition 4

**Lemma** **A1.**
*Let ${\left\{B_{j}\right\}}_{j=1}^{\ell }$ (with ℓ≥2) be a sequence of sets that is not a chain (i.e., there is no permutation π:[ℓ]→[ℓ] such that $B_{\pi (1)}\subseteq B_{\pi (2)}\subseteq \ldots \subseteq B_{\pi (\ell )}$). Consider a recursive process where, at each step, a pair of sets that are not related by inclusion is replaced with their intersection and union. Then, there exists such a recursive process that leads to a chain in a finite number of steps.*

**Proof.** The lemma is proved by mathematical induction on *ℓ*. It holds for ℓ=2 since $B_{1}\cap B_{2}\subseteq B_{1}\cup B_{2}$, and the process halts in a single step. Suppose that the lemma holds with a fixed ℓ≥2, and for an arbitrary sequence of *ℓ* sets which is not a chain. We aim to show that it also holds for every sequence of ℓ+1 sets which is not a chain. Let ${\left\{B_{j}\right\}}_{j=1}^{\ell +1}$ be such an arbitrary sequence of sets, and consider the subsequence of the first *ℓ* sets $B_{1},\ldots ,B_{\ell }$. If it is not a chain, then (by the induction hypothesis) there exists a recursive process as above which enables to transform it into a chain in a finite number of steps, i.e., we get a chain $B_{1}^{\prime }\subseteq B_{2}^{\prime }\subseteq \ldots \subseteq B_{\ell }^{\prime }$. If $B_{\ell }^{\prime }\subseteq B_{\ell +1}$ or $B_{\ell +1}\subseteq B_{1}^{\prime }$, then we get a chain of ℓ+1 sets. Otherwise, by proceeding with the recursive process where $B_{\ell }^{\prime }$ and $B_{\ell +1}$ are replaced with their intersection and union, consider the sequence

(A15) $$\begin{array}{c} B_{1}^{\prime },\;\ldots ,\;B_{\ell -1}^{\prime },\;B_{\ell }^{\prime }\cap B_{\ell +1},\;B_{\ell }^{\prime }\cup B_{\ell +1}. \end{array}$$

By the induction hypothesis, the first *ℓ* sets in this sequence can be transformed into a chain (in a finite number of steps) by a recursive process as above; this gives a chain of the form $B_{1}^{\prime \prime }\subseteq B_{2}^{\prime \prime }\ldots \subseteq B_{\ell -1}^{\prime \prime }\subseteq B_{\ell }^{\prime \prime }$. The first *ℓ* sets in (A15) are all included in $B_{\ell }^{\prime }$, so every combination of unions and intersections of these *ℓ* sets is also included in $B_{\ell }^{\prime }$. Hence, the considered recursive process leads to a chain of the form

(A16) $$\begin{array}{c} B_{1}^{\prime \prime }\subseteq B_{2}^{\prime \prime }\ldots \subseteq B_{\ell -1}^{\prime \prime }\subseteq B_{\ell }^{\prime \prime }\subseteq B_{\ell }^{\prime }\cup B_{\ell +1}, \end{array}$$

where the last inclusion in (A16) holds since $B_{\ell }^{\prime \prime }\subseteq B_{\ell }^{\prime }$. The claim thus holds for ℓ+1 if it holds for a given *ℓ*, and it holds for ℓ=2, it therefore holds by mathematical induction for all integers ℓ≥2. □

We first prove Proposition 4a. Suppose that there is a permutation π:[M]→[M] such that $S_{\pi (1)}\subseteq S_{\pi (2)}\subseteq \ldots \subseteq S_{\pi (M)}$ is a chain. Since every element in Ω is included in at least *d* of these subsets, then it should be included in (at least) the *d* largest sets of this chain, so $S_{\pi (j)}=\Omega$ for every j∈[M−d+1:M]. Due to the non-negativity of *f*, it follows that

(A17a)∑j=1Mf(Sj)≥∑j=M−d+1Mf(Sπ(j))(A17b)=df(Ω).

Otherwise, if we cannot get a chain by possibly permuting the subsets in the sequence $\{S_{j}{\}}_{j=1}^{M}$, consider a pair of subsets $S_{n}$ and $S_{m}$ that are not related by inclusion, and replace them with their intersection and union. By the submodularity of *f*,

(A18a)∑j=1Mf(Sj)=∑j≠n,mf(Sj)+f(Sn)+f(Sm)(A18b)≥∑j≠n,mf(Sj)+f(Sn∩Sm)+f(Sn∪Sm).

For all ω∈Ω, let deg(ω) be the number of indices j∈[M] such that $\omega \in S_{j}$. By replacing $S_{n}$ and $S_{m}$ with $S_{n}\cap S_{m}$ and $S_{n}\cup S_{m}$, the set of values ${\left\{\deg\left(\omega \right)\right\}}_{\omega \in \Omega }$ stays unaffected (indeed, if $\omega \in S_{n}$ and $\omega \in S_{m}$, then it belongs to their intersection and union; if ω belongs to only one of the sets $S_{n}$ and $S_{m}$, then $\omega \notin S_{n}\cap S_{m}$ and $\omega \in S_{n}\cup S_{m}$; finally, if $\omega \notin S_{n}$ and $\omega \notin S_{m}$, then it does not belong to their intersection and union). Now, consider the recursive process in Lemma A1. Since the profile of the number of inclusions of the elements in Ω is preserved in each step of the recursive process in Lemma A1, it follows that every element in Ω stays to belong to at least *d* sets in the chain which is obtained at the end of this recursive process. Moreover, in light of (A18), in every step of the recursive process in Lemma A1, the sum in the LHS of (A18) cannot increase. Inequality (45) therefore finally follows from the earlier part of the proof for a chain (see (A17)).

We next prove Proposition 4b. Let A⊂Ω, and suppose that every element in A is included in at least d≥1 of the subsets ${\left\{S_{j}\right\}}_{j=1}^{M}$. For all j∈[M], define $S_{j}^{\prime }≜S_{j}\cap A$, and consider the sequence ${\left\{S_{j}^{\prime }\right\}}_{j=1}^{M}$ of subsets of A. If *f* is a rank function, then it is monotonically increasing, which yields

(A19) $$\begin{array}{c} f\left(S_{j}^{\prime }\right)\leq f\left(S_{j}\right),\;j\in \left[M\right]. \end{array}$$

Each element of A is also included in at least *d* of the subsets ${\left\{S_{j}^{\prime }\right\}}_{j=1}^{M}$ (by construction, and since (by assumption) each element in A is included in at least *d* of the subsets ${\left\{S_{j}\right\}}_{j=1}^{M}$). By the non-negativity and submodularity of *f*, Proposition 4a gives

(A20) $$\begin{array}{c} \sum_{j=1}^{M}f\left(S_{j}^{\prime }\right)\geq d\;f\left(A\right). \end{array}$$

Combining (A19) and (A20) yields (46). This completes the proof of Proposition 4.

**Remark** **A1.**
*Lemma A1 is weaker than a claim that, in every recursive process as in Lemma A1, the number of pairs of sets that are not related by inclusion is strictly decreasing at each step. Lemma A1 is, however, sufficient for our proof of Proposition 4a.*

## References

[1] Cover T.M., Thomas J.A. (2006). Elements of Information Theory.

[2] Dembo A., Cover T.M., Thomas J.A. (1991). Information theoretic inequalities. IEEE Trans. Inf. Theory.

[3] Chan T. (2011). Recent progresses in characterising information inequalities. Entropy.

[4] Martin S., Padró C., Yang A. (2016). Secret sharing, rank inequalities, and information inequalities. IEEE Trans. Inf. Theory.

[5] Babu S.A., Radhakrishnan J., Agrawal M., Arvind V. (2014). An entropy-based proof for the Moore bound for irregular graphs. Perspectives on Computational Complexity.

[6] Boucheron S., Lugosi G., Massart P. (2013). Concentration Inequalities - A Nonasymptotic Theory of Independence.

[7] Chung F.R.K., Graham L.R., Frankl P., Shearer J.B. (1986). Some intersection theorems for ordered sets and graphs. J. Comb. Theory Ser. A.

[8] Erdos P., Rényi A. (1963). On two problems of information theory. Publ. Math. Inst. Hung. Acad. Sci..

[9] Friedgut E. (2004). Hypergraphs, entropy and inequalities. Am. Math. Mon..

[10] Jukna S. (2011). Extremal Combinatorics with Applications in Computer Science.

[11] Kaced T., Romashchenko A., Vereshchagin N. (2018). A conditional information inequality and its combinatorial applications. IEEE Trans. Inf. Theory.

[12] Kahn J. (2001). An entropy approach to the hard-core model on bipartite graphs. Comb. Comput..

[13] Kahn J. (2001). Entropy, independent sets and antichains: A new approach to Dedekind’s problem. Proc. Am. Math. Soc..

[14] Madiman M., Marcus A.W., Tetali P. (2012). Entropy and set cardinality inequalities for partition-determined functions. Random Struct. Algorithms.

[15] Madiman M., Marcus A.W., Tetali P. Information–theoretic inequalities in additive combinatorics. Proceedings of the 2010 IEEE Information Theory Workshop.

[16] Pippenger N. (1977). An information–theoretic method in combinatorial theory. J. Comb. Ser. A.

[17] Pippenger N. (1999). Entropy and enumeration of boolean functions. IEEE Trans. Inf. Theory.

[18] Radhakrishnan J. (1997). An entropy proof of Bregman’s theorem. J. Comb. Theory Ser. A.

[19] Radhakrishnan J. (2001). Entropy and counting. Computational Mathematics, Modelling and Algorithms.

[20] Sason I. (2021). A generalized information–theoretic approach for bounding the number of independent sets in bipartite graphs. Entropy.

[21] Sason I. Entropy-based proofs of combinatorial results on bipartite graphs. Proceedings of the 2021 IEEE International Symposium on Information Theory.

[22] Madiman M., Tetali P. (2010). Information inequalities for joint distributions, interpretations and applications. IEEE Trans. Inf. Theory.

[23] Bach F. (2013). Learning with submodular functions: A convex optimization perspective. Found. Trends Mach. Learn..

[24] Chen Q., Cheng M., Bai B. (2021). Matroidal entropy functions: A quartet of theories of information, matroid, design and coding. Entropy.

[25] Fujishige S. (1978). Polymatroidal dependence structure of a set of random variables. Inf. Control..

[26] Fujishige S. (2005). Submodular Functions and Optimization.

[27] Iyer R., Khargonkar N., Bilems J., Asnani H. (2022). Generalized submodular information measures: Theoretical properties, examples, optimization algorithms, and applications. IEEE Trans. Inf. Theory.

[28] Krause A., Guestrin C. Near-optimal nonmyopic value of information in graphical models. Proceedings of the Twenty-First Conference on Uncertainty in Artificial Intelligence (UAI 2005).

[29] Lovász L., Bachem A., Korte B., Grotschel M. (1983). Submodular functions and convexity. Mathematical Programming The State of the Art.

[30] Tian C. Inequalities for entropies of sets of subsets of random variables. Proceedings of the 2011 IEEE International Symposium on Information Theory.

[31] Kishi Y., Ochiumi N., Yanagida M. Entropy inequalities for sums over several subsets and their applications to average entropy. Proceedings of the 2014 IEEE International Symposium on Information Theory (ISIT 2014).

[32] Shannon C.E. (1948). A Mathematical Theory of Communication. Bell Syst. Tech. J..

[33] Madiman M., Mellbourne J., Xeng P., Carlen E., Madiman M., Werner E.M. (2017). Forward and reverse entropy power inequalities in convex geometry. Convexity and Concentration.

[34] Han T.S. (1978). Nonnegative entropy measures of multivariate symmetric correlations. Inf. Control..

[35] Polyanskiy Y., Wu Y. Lecture Notes on Information Theory, version 5. http://people.lids.mit.edu/yp/homepage/data/itlectures_v5.pdf.

[36] Bollobás B. (1978). Extremal Graph Theory.

[37] Madiman M. On the entropy of sums. Proceedings of the 2008 IEEE Information Theory Workshop.

[38] Tao T. (2010). Sumset and inverse sumset theory for Shannon entropy. Comb. Comput..

[39] Kontoyiannis I., Madiman M. (2014). Sumset and inverse sumset inequalities for differential entropy and mutual information. IEEE Trans. Inf. Theory.

[40] Artstein S., Ball K.M., Barthe F., Naor A. (2004). Solution of Shannon’s problem on the monotonicity of entropy. J. Am. Soc..

[41] Madiman M., Barron A. (2007). Generalized entropy power inequalities and monotonicity properties of information. IEEE Trans. Inf. Theory.

